# Physiological and Psychological Assessments for the Establishment of Evidence-Based Forest Healing Programs

**DOI:** 10.3390/ijerph18179283

**Published:** 2021-09-02

**Authors:** Sujin Park, Yeji Choi, Geonwoo Kim, Eunsoo Kim, Soojin Kim, Domyung Paek

**Affiliations:** 1 Future Forest Strategy Department, Forest Human Service Division, National Institute of Forest Science, Seoul 02455, Korea; snowshoe@korea.kr (S.P.); usmile.choi@gmail.com (Y.C.); bkim5020@korea.kr (G.K.); euncarp2@gmail.com (E.K.); kimsoojinsj@korea.kr (S.K.); 2 Graduate School of Public Health, Seoul National University, Seoul 08826, Korea; 3 Institute of Health and Environment, Seoul National University, Seoul 08826, Korea

**Keywords:** forest healing, forest healing program, forest therapy, physiological effect, psychological effect, follow-up survey, long-term observation

## Abstract

This study aimed to establish a health and medical foundation for forest healing programs and provide a basis for developing an evaluation system for such programs. While the number of visitors to forests and interest in forest healing effects are increasing, few studies have examined the various indicators of the persistent changes in forest healing effects. Therefore, this study conducted pre-, post-, and follow-up experiments on 87 health and clinical indicators in a sample of 88 adolescent participants. The relationships between pre-, post-, and follow-up experiment results for each indicator were analyzed. Of the 87 indicators, 46 showed significant changes, including systolic blood pressure, diastolic blood pressure, cholesterol, serotonin, vitamin D, CD16+CD56 count, interferon-γ, resilience, and self-esteem. The findings are significant for studying diverse participants and indicators and lay the foundation for developing forest healing programs by clarifying aspects such as the indicators suitable for short-term observation versus the indicators requiring long-term observation. Based on these analyses, the results of this study are expected to be useful when conducting research to establish an evidence-based forest healing program in the future.

## 1. Introduction

### 1.1. Research Background and Rationale

Rapid industrialization and urbanization have led to a decline in the quality of life and happiness of urban residents, and forest healing programs are drawing attention as a means of recharging life energy and improving health [1]. Increasingly, more research is examining the health and preventive medical effects of green space, such as reducing stress and preventing diseases [2,3,4,5,6,7,8,9]. With growing interest in verifying the forest healing effects of green spaces, various studies are also underway to investigate the physiological and psychological effects of forest healing. Several studies have demonstrated that forest healing has a positive effect on various diseases [10,11,12,13,14,15]. In fact, a systematic review and meta-analysis of 143 studies showed that green space and forest healing programs have positive effects on various aspects of human physiological health, including a reduction in blood pressure, cortisol, and heart rate, and showed positive outcomes for neurological diseases, cancer, and respiratory-related mortality rates [16]. Another study demonstrated that forest healing not only improved physical health indicators, but also improved the immune system through NK cell activation, resulting in preventive medical effects even after the end of the program [17].

In addition to physiological health, several studies are underway to identify the positive effects of forest healing on psychological health [15,18,19,20,21,22,23,24,25,26]. A prior systematic review concluded that forest healing programs greatly helped alleviate depression [18]. Furthermore, other studies have found that forest healing programs help relieve anxiety and depression [19]; positively affect mood, boost vitality, and reduce negative emotions such as tension anxiety, depression, anger, hostility, fatigue, and confusion [23]; and reflect on participants’ values, leading to positive psychological change [26]. As such, forest healing has a positive effect on human physiological and psychological health, but the exact mechanisms that help promote them are not yet known [27].

Several prior studies have categorized forest healing effects into olfactory, visual, and environmental effects with social engagement. The olfactory effect of forest healing through green space exposure is caused by monoterpenes that radiate from forests and trees. Monoterpene is a volatile substance found in flowers and trees that functions as a sterilization agent and insecticide. α-Pinene accounts for most monoterpenes produced in forests, and several prior studies have demonstrated its anti-inflammatory, antioxidant, and anti-anxiety effects [28,29,30]. In addition, several monoterpenes, including limonene and β-pinene, have been proven to yield various positive outcomes, including enhancing the immune system, relieving cardiovascular disease, and improving depression [31,32,33,34,35,36,37]. Depending on the type of forests and the species that make up the forests, it is known that the amount and type of monoterpene emitted differs. Generally, higher concentrations of monoterpene are produced in coniferous forests than in broadleaf forests [38,39,40,41]. 

The positive physical changes achieved simply by looking at forests and trees represent the visual component of the forest healing effect. An experiment demonstrated positive psychological and physiological changes after viewing natural photographs among participants who were shown natural and urban photographs [42,43,44,45,46]. This indicates that even the indirect experience of looking at pictures of forests has effective forest healing outcomes. 

Finally, regarding the environmental effects with social engagement, a study showed that the top 20% of the population in terms of the green space rate within a radius of 250 m around their home had a 12% lower mortality rate than those in the bottom 20%; the difference was most pronounced in respiratory and cancer-related mortality [47]. In addition, the green space rate near the residence was found to have a positive effect in improving mental health, including depression [27]. Researchers have identified the social and environmental functions of green spaces as a cause of this positive relationship between green spaces and health. Green spaces around the house increase residents’ exercise activities and social engagements and decrease stress; additionally, air and noise pollution are reduced. The improved surroundings and increased exercise activities promote physical health, and increased social relationships and reduced stress have a positive effect on psychological health [27,48,49]. As mentioned earlier, the mechanisms underlying forest healing effects have not yet been accurately identified; however, based on prior studies, research on the impact of forest healing on individual human health indicators is actively underway.

Most prior studies on the impact of forest healing on individual health indicators have focused on short-term evaluations, comparing pre- and post-test results on individual health indicators. However, comprehensive evaluations using diverse health and clinical indicators have not been conducted. In addition, although prior studies have analyzed the physiological and psychological effects of forest healing programs, few studies have investigated the long-term maintenance of forest healing effects through follow-up examination after the program ends. To establish a health and medical foundation for forest healing, continuous and repeated research is needed. However, at present, there is a lack of evidence to establish an evaluation system for forest healing. As the number of visitors heading to the forest for healing is rapidly growing, and there is increasing curiosity about the effectiveness of forest healing programs, it is urgent to establish a medical basis to prove the effectiveness of forest healing.

### 1.2. Research Purpose and Questions

This study was conducted to comprehensively evaluate the physiological and psychological effects of forest healing, based on health and clinical indicators, as a pilot attempt to lay the foundation for a forest healing program. This study aimed to verify the effectiveness of forest healing and to confirm the sustainability and change in forest healing effects through pre-, post-, and follow-up tests. In addition, the experimental period and measurements were applied on a trial basis considering the various indicators and measurement factors. Therefore, this study is meaningful in providing a basis for future research and developing an evidence-based forest healing program.

The following research questions were addressed: Is there a correlation between green space exposure level and health conditions? What are the indicators of distinct changes during the study period? Which indicators are resistant to change, and which indicators are more susceptible to change? Which indicators are appropriate for short-term evaluation, and which indicators require long-term observation? 

## 2. Materials and Methods

### 2.1. Research Design

**Figure 1:**
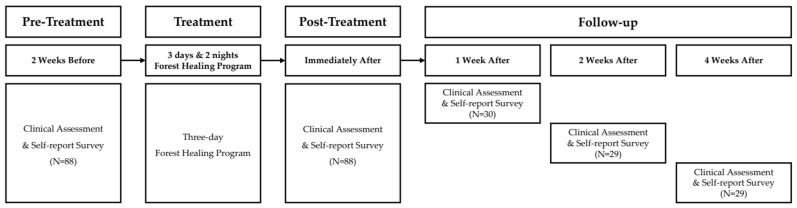
Schematic diagram of the research design.

To investigate the physiological and psychological effects of the forest healing program, 92 participants were recruited for the program lasting three days and two nights. Two weeks before the commencement of the forest healing program, the participants underwent a clinical assessment and responded to a self-report survey; the same assessments were conducted again shortly after the program ended. Post-treatment was measured immediately after the end of the program, but the timing of the individual participants’ measurements slightly differed due to the large number of participants. All post-treatment measurements were completed within one hour after the program. After the program, the participants were divided into three groups, and the sustainability of the changes after returning to their daily lives was assessed using the same assessment measures as pre-test and post-test after one, two, and four weeks in Groups 1, 2, and 3, respectively. A schematic diagram of the research design is presented in Figure 1.

### 2.2. Participants

The study was conducted with 92 adolescents aged 13–18 years, in July and August 2017, supported by the Jung Mong-gu Foundation hosted by the Korea Forest Welfare Promotion Agency. Participants were youth in residential care facilities who lived in a group in one space, and they were from the following three cities: Deajeon (*n* = 32), Gimcheon (*n* = 29), and Incheon (*n* = 29). After excluding two participants who dropped out of the study and one who got injured during the research period, the final sample comprised 88 participants (30 from Daejeon and 29 each from Gimcheon and Incheon).Informed consent was obtained from the participants and their guardians after clearly explaining the purpose and objectives of the research. The study was conducted in accordance with the guidelines of the Declaration of Helsinki, and the protocol was approved by the Wonkwang University Institutional Review Board (protocol code WKIRB-201705-BM-027). The demographic characteristics of the participants are presented in Table 1.

The normalized difference vegetation index (NDVI) of the regions, Gimcheon, Daejeon, and Incheon, was examined; the results are shown in Table 2. NDVI was calculated based on the shelters where the participants lived. Gimcheon was found to have the largest distribution of green areas in both the living area and areas within walking distance; the green area rate of Daejeon’s living area was significantly lower than that of other areas, and Incheon had the lowest green area rate in walking distance.

### 2.3. Sites

**Figure 2:**
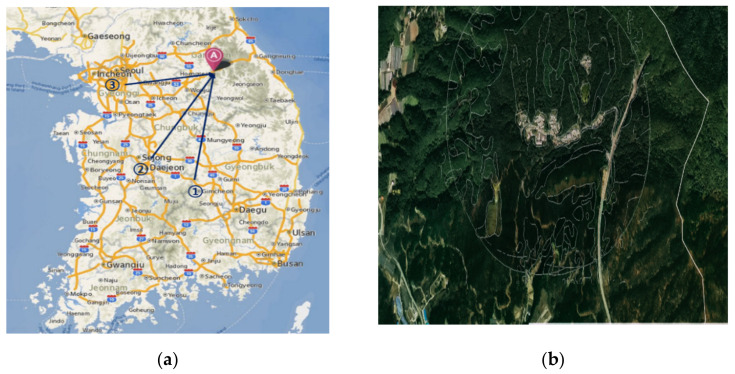
Maps of the study site. (**a**) Site location. Point A is Heongseong SoopChewon, and Points 1, 2, and 3 are the three cities from where the participants came. (**b**) Aerial photographs of Heongseong SoopChewon.

The forest healing program was conducted at Heongseong SoopChewon, Gangwon, Korea. It is a national forest park located 840 m above sea level on Cheong-Tae Mountain (Figure 2). Heongseong SoopChewon provides forest welfare services that enhance human immunity and restore physical and mental health by utilizing various environmental factors and natural objects in the forest. Heongseong SoopChewon was designated as the first forest education center in Korea in September 2007, and it actively provides forest education and healing programs for youth and other participants. 

The percentage of forest area within walking distance of the study site was 92.24%, and the crown density was 79.20% (Table 3). The average NDVI was 0.392. The adjacent forest included eight types of vegetation communities, including *Larix kaempferi*, *Quercus aliena, Pinus densiflora, Abies holophylla, Betula platyphylla, Pinus Koraiensis* community, and deciduous broad-leaved and mixed communities. The widest site was *the Larix kaempferi* community (69.37%). 

### 2.4. Treatment

The three-day and two-night forest healing program was conducted in the forest. The program utilized various forest resources within Heongseong SoopChewon, such as free walking in the forest, recreation in the woods, and woodworking experience (Table 4). The participants were divided into three groups that came from differing regions. The participants did not undergo the program simultaneously: they all completed the program within an interval of two to three days. All groups partook in the same program in the same location to guarantee their experiences were indistinguishable from each other. All activities were organized and conducted in groups of 15, and they were minimally guided by instructors. The locations and contents of the program were explained to them, but they were free to independently move around and decide what to create if it fits the program goals. Except for orientation, lectures and post-test measurement, the entirety of the program took place in forests.

### 2.5. Measurement

The measurement factors were divided into the following three main categories: self-report survey, clinical assessment, and qualitative assessment. As shown in Table 5, the self-report survey included an effectiveness evaluation indicating resilience, interpersonal competency, self-esteem, stress response, and vigor. Clinical assessment included physical examination, complete blood count, biochemical examination, immunoserological examination, saliva test, and urine test. The main indicators among the total 87 indicators are listed in Table 5. Lastly, the qualitative assessment that was conducted right after the program included participant interviews, including focus group interviews and individual interviews, as well as teacher interviews with forest education experts and program guidance teachers. 

### 2.6. Analysis 

**Figure 3:**
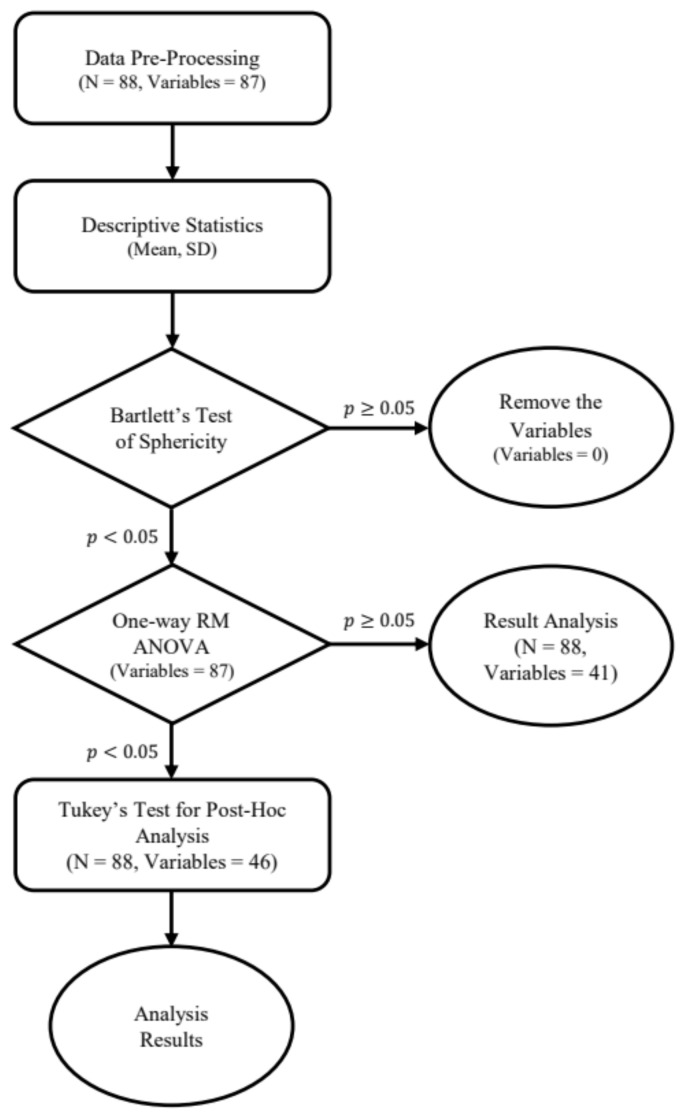
Data analysis process diagram.

The changes in the indicators between pre-test, post-test, and follow-up were analyzed for a total of 88 participants (male = 49, female = 39), who were tested three times during the study period. A total of 87 variables, including 82 physiological indicators and 5 psychological indicators were examined using R 4.0.3 version and R Studio to calculate descriptive statistics and one-way repeated measures ANOVA (Figure 3). All values were rounded to the fifth decimal place, and normality was verified using the central limit theorem. Bartlett’s test of sphericity was performed before the one-way repeated measures ANOVA. Subsequently, Tukey’s test for post hoc analysis was conducted on 46 indicators with a significance level of *p* < 0.05, to identify the significant differences determined using the ANOVA.

## 3. Results

Of the 87 health-related indicators analyzed, 46 indicators showed significant differences in one-way repeated measures ANOVA, and 28 indicators showed significant differences in Tukey’s test for post hoc analysis. The results of ANOVA and post hoc tests of all indicators are presented in Appendix A and Appendix B. The results of this study are described based on a statistical analysis of the 88 people as mentioned above, and the results of ANOVA analysis for each group can be found in Appendix C.

### 3.1. Blood Pressure and Autonomic Nervous System

**Figure 4:**
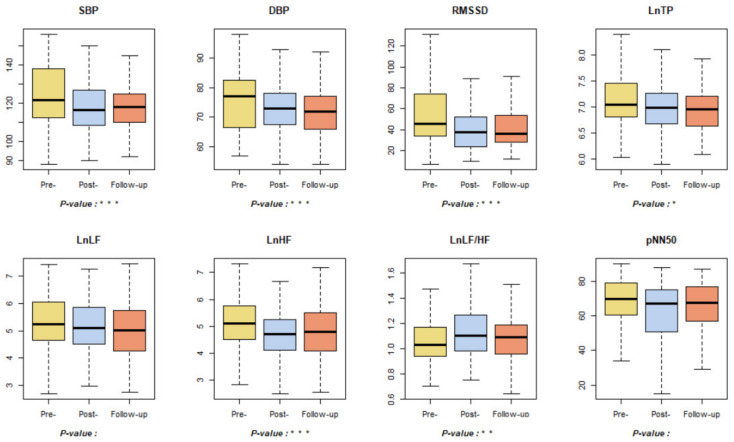
Results of blood pressure and autonomic nervous system related indicators. * *p* < 0.05, ** *p* < 0.01, *** *p* < 0.001. Pre-: daily health conditions two weeks prior to the forest healing program; Post-: health conditions immediately after the forest healing program; Follow-up: daily health conditions one, two, and four weeks after the forest healing program; SBP: systolic blood pressure; DBP: diastolic blood pressure; RMSSD: root mean square of successive RR intervals (interbeat intervals between all successive heartbeats) differences; Ln: natural logarithm (the spectral power data were log transformed); TP: total power; LF: power in the low frequency range; HF: power in the high frequency range; pNN50: percentage of successive RR intervals that differ by more than 50 ms.

Cardiovascular-related indicators showed significant reductions in systolic and diastolic blood pressure and cholesterol levels in the post-test, consistent with prior studies showing significant reductions in blood pressure after forest healing programs as shown in Figure 4 [17,20,50,51]. The diastolic blood pressure decreased significantly in the follow-up test, but systolic blood pressure and cholesterol tended to increase in follow-up tests. This is in line with the results of prior studies that conducted an assessment more than a week after the forest healing program and reported that the effect of forest healing had been reduced within three to five days [20,50,51].

The analysis of the heart rate variability (HRV) indicators influencing stress responses showed that LnTP and RMSSD decreased significantly over the pre-, post-, and follow-up tests, and LnLF also tended to decrease over the pre-, post-, and follow-up tests, although not significantly. LnHF decreased significantly in the post-test compared to the pre-test but increased in the follow-up test. LnLF/LnHF significantly increased during the post-test but decreased during the follow-up test. As such, the increased LnHF and the decreased LnLF/LnHF levels after the forest healing program are consistent with the results of prior studies and considering that the effect could be observed in the follow-up test, not in the post-test, it is expected that the effect takes some time to appear [10,50]. Although no significant results were observed for pNN50, there was a significant decrease in the post-test compared to the pre-test in male participants. The results of the analysis of HRV indicators were generally insignificant or negative, and within the normal range. Further studies need to be conducted for the same.

**Figure 5:**
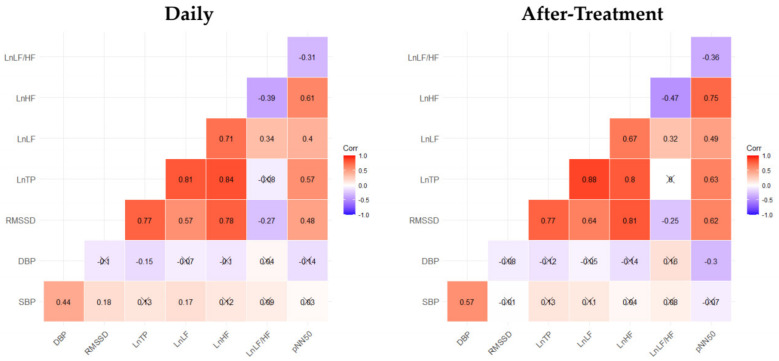
Pearson’s correlation coefficient results for blood pressure and autonomic nervous system related indicators. Cells marked with X indicate that the correlation coefficient is not significant (*p* > 0.05). Daily analysis includes pre-treatment and follow-up test results, and after-treatment analysis includes post-treatment results.

Blood pressure and HRV levels are known to affect each other, and diastolic blood pressure is more closely related to the autonomic nervous system than systolic blood pressure [52,53]. Previous studies have shown that diastolic blood pressure is inversely correlated with RMSSD, LF, HF, and TP, and positively correlated with LF/HF. This is consistent with the results of this study, and although the diastolic blood pressure-related correlation analysis in this study did not show significant results for all of the above HRV indicators, the results confirmed that they tend to be the same as in prior studies as shown in Figure 5 [53,54]. However, in terms of systolic blood pressure, the present results differed from the previous literature; therefore, follow-up research is needed. In addition, there were strong positive linear correlations among the HRV-related indicators, except for LnLF/HF, and a significant positive correlation between systolic and diastolic blood pressure. A slightly stronger linear relationship in general was observed in the after-treatment results than in everyday conditions, including pre-treatment and follow-up results.

### 3.2. Immune Function and Inflammation

When examining immune-related indicators, both CD16+CD56 (count) and CD16+CD56 (WB), which affect the activity of NK cells, decreased in the post-test compared to the pre-test, but increased significantly in the follow-up test. This result is consistent with prior studies showing that forest healing effectively increases NK cell activity and population [17,28,55]. In cytokine analysis, IFN-γ significantly increased in the pre-, post-, and follow-up tests, and IFN-γ is known to enhance immunity by activating NK cells and macrophages. Whereas previous studies have not identified significant changes in IFN-γ through forest healing programs, the results show that forest healing programs have a positive impact on anti-cancer and immune systems through an increase in IFN-γ [56]. IL-4 did not show significant changes, and IL-8 increased abnormally after the forest healing program in Groups one and two. This is because IL-8 is closely related to the allergic inflammatory reaction, and IL-8 levels increase in such cases [3,57,58]. Groups one and two showed a significantly higher sensitization to allergic antigens compared to Group three, and IL-8 levels showed a higher allergic sensitization rate in the group with a post-treatment increase than in the group with a post-treatment decrease compared to the pre-treatment (Appendix D). In particular, Group one showed a very high number of participants with plant-related allergic antigens compared to the other groups [59,60]. In the case of TNF-α, significant reductions were observed across the pre-, post-, and follow-up phases. As TNF-α causes a strong inflammatory response, the reduction in TNF-α indicates that forest healing programs help suppress inflammatory responses [61].

The indicator associated with inflammation, eNO, showed no significant difference, and the analysis of white blood cells (WBC) showed that the total number of WBC and basophils decreased in post-test compared to pre-test, but significantly increased in the follow-up test. Lymphocytes and monocytes tended to decrease over pre-, post-, and follow-up tests, in contrast to a prior study that observed significant increases in lymphocytes and monocytes [62,63]. The reason for this difference is that the proportion and total number of WBC elements, including lymphocytes, vary depending on age group; this study was conducted on adolescents, whereas the previous study was conducted on participants in their mid-40s.

**Figure 6:**
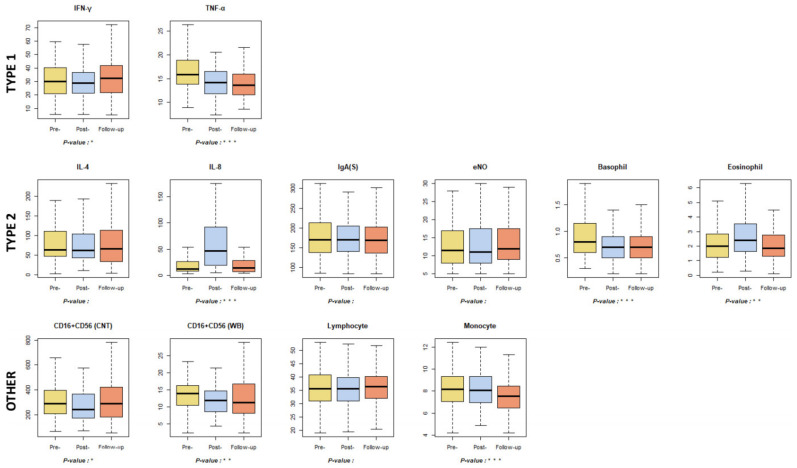
Results of immune function and inflammation-related indicators. * *p* < 0.05, ** *p* < 0.01, *** *p* < 0.001. Pre-: daily health conditions two weeks prior to the forest healing program; Post-: health conditions immediately after the forest healing program; Follow-up: daily health conditions one, two, and four weeks after the forest healing program; IFN: interferon; TNF: tumor necrosis factor; IL: interleukin; IgA: Immunoglobulin A; eNO: exhaled nitric oxide; CD: cluster of differentiation molecule.

Human immune function is achieved through the balance of two types of helper T cells, Th1 (Type 1) and Th2 (Type 2). Th1 is a regulator of cell-mediated immunity, which increases inflammation in infected cells; improves macrophage function in response to viral and bacterial infections; and plays a role in innate immunity, and elimination of infectious substances. [64,65,66,67,68,69]. Th2 is a modulator of humoral immunity, which improves the function of eosinophils, basophils, and mast cells to counteract parasitic infections and is immune to antigen antibody responses [64,68,69]. Among the indicators observed in this study, those corresponding to Th1 and Th2 cells are shown in Figure 6. If Th1 increases, autoimmune disease is known to increase; enhanced Th2 indicates the presence of allergic diseases [64,65,66,67,68,69]. Most of the indicators of Th1 and Th2 tracked in this study showed reductions after the forest healing program, which can be interpreted as a reduction in autoimmune and allergic diseases.

### 3.3. Oxidative Stress and Antioxidant

**Figure 7:**
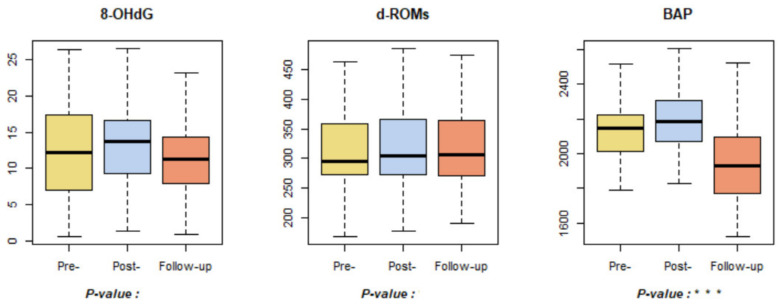
Results of oxidative stress and antioxidant-related indicators. *** *p* < 0.001. Pre-: daily health conditions 2 weeks prior to the forest healing program; Post-: health conditions immediately after the forest healing program; Follow-up: daily health conditions one, two, and four weeks after the forest healing program; 8-OHdG: 8-hydroxy-2-deoxyguanosine; d-ROMs: derivatives of reactive oxygen metabolites; BAP: biological antioxidant potential.

The analysis of 8-OHdG and d-ROMs, indicators related to oxidative stress, showed minimal changes (Figure 7). Although there is a lack of prior research on these indicators, based on the results of this study, it can be concluded that the forest healing program had a minimal impact on 8-OHdG and d-ROMs. BAP, an antioxidant-related indicator, increased significantly in post-test compared to pre-test, and decreased in the follow-up tests. This is consistent with the results of a previous study, where antioxidant levels increased significantly immediately after the forest healing program [70]. However, although the prior study did not conduct a further investigation into antioxidant functions, this study shows that antioxidant levels decreased again after a certain period of time had elapsed after the forest healing program.

### 3.4. Stress (Hormone)

**Figure 8:**
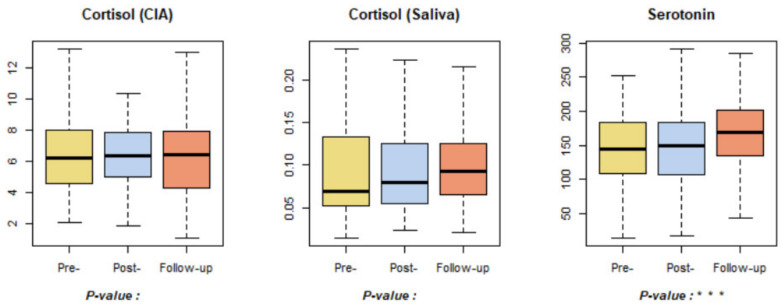
Results of stress (hormone)-related indicators. *** *p* < 0.001. Pre-: daily health conditions two weeks prior to the forest healing program; Post-: health conditions immediately after the forest healing program; Follow-up: daily health conditions one, two, and four weeks after the forest healing program.

Neither cortisol (CIA) nor the cortisol (saliva) related to stress hormones showed significant changes (Figure 8). This result is in contrast to a previous study, which found that forest healing experiences had a positive effect on stress control by reducing cortisol levels [7]. However, the previous study was conducted with middle-aged women; it is expected that the effects were not apparent in this study because the participants were adolescents. Serotonin levels increased significantly over the course of the pre-, post-, and follow-up tests, which is consistent with the results of a previous study that showed significant increases in serotonin levels after the forest healing program [14].

### 3.5. Health Screening Parameters

**Figure 9:**
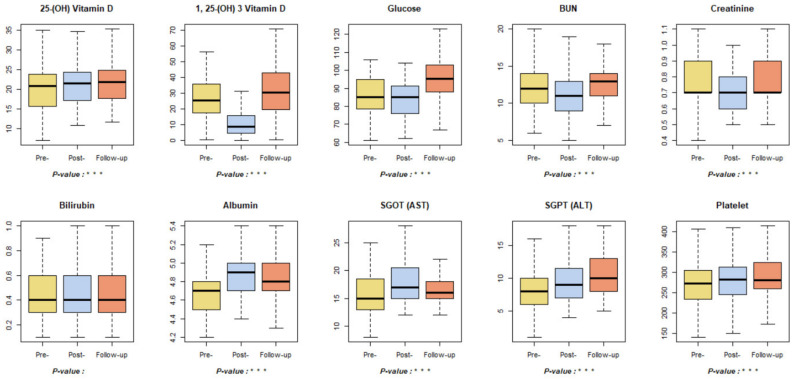
Results of health screening-related indicators. *** *p* < 0.001. Pre-: daily health conditions two weeks prior to the forest healing program; Post-: health conditions immediately after the forest healing program; Follow-up: daily health conditions one, two, and four weeks after the forest healing program; BUN: blood urea nitrogen; SGOT: serum glutamic oxaloacetic transaminase; AST: aspartate aminotransferase; SGPT: serum glutamic pyruvic transaminase; ALT: alanine aminotransferase.

Among the health screening indicators, 25-(OH) vitamin D increased significantly over the pre-, post-, and follow-up tests, while 1,25-(OH) 3 vitamin D decreased significantly during the post-test compared to the pre-test and increased again during the follow-up test (Figure 9). Glucose, an indicator of obesity, reduced significantly during the post-test, and increased during the follow-up test. A systematic review of the effects of forest healing observed a decrease in glucose levels immediately after a forest healing program; however, it was limited by the inability to demonstrate a continuous trend in blood sugar levels [71,72]. The results of the glucose levels obtained in this study demonstrate that blood sugar levels do not show a continuous trend of decline and tend to increase after forest healing. The BUN and creatine levels associated with renal function significantly decreased in the post-test compared to the pre-test and increased in the follow-up examinations. This is in the same vein as a prior study that demonstrated improved kidney function through forest healing programs, although the improvement was short-lived [7]. Among the indicators associated with liver function, bilirubin showed no significant results, and albumin and SGOT (AST) increased significantly in the post-test but decreased significantly in the follow-up test. The SGPT (ALT) and platelet counts increased significantly throughout the pre-, post-, and follow-up periods. As such, liver function was difficult to analyze due to a mixture of positive and negative results, but all the indicators changed within normal limits.

### 3.6. Mental Health

**Figure 10:**
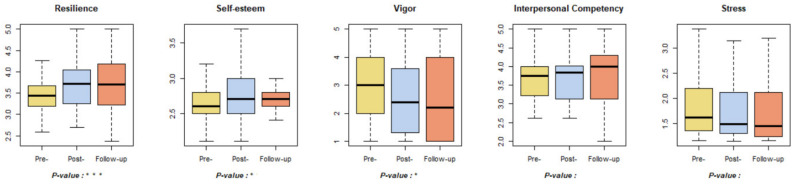
Results of mental health-related indicators. * *p* < 0.05, *** *p* < 0.001. Pre-: daily health conditions two weeks prior to the forest healing program; Post-: health conditions immediately after the forest healing program; Follow-up: daily health conditions one, two, and four weeks after the forest healing program.

Among the psychological indicators, significant results were observed for resilience, self-esteem, and vigor (Figure 10). Resilience increased significantly in the post-test compared to the pre-test but decreased in the follow-up test. Self-esteem increased through the pre-, post-, and follow-up tests. On the other hand, vigor decreased through the pre-, post-, and follow-up tests. Further studies on the degradation of vigor levels are needed, as a systematic review suggests that forest healing has a positive effect on vigor [73].

## 4. Discussion

The forest healing program led to positive changes in several indicators. Of the 87 indicators, 46 showed significant changes, including systolic blood pressure, diastolic blood pressure, cholesterol, serotonin, vitamin D, CD16+CD56 count, IFN-γ, resilience, and self-esteem. IL-8 has been shown to be susceptible to interpersonal deviations, and cholesterol and BAP are considered appropriate for short-term effect observations. Long-term observation is required for indicators such as blood pressure, TNF-α, IFN-γ, and serotonin, for which effects were shown to last till the follow-up assessment, as well as for indicators such as CD16+CD56 (count, WB), IL-4, vitamin D, and interpersonal competency, for which the effects were not expressed immediately after the program but were observed in the follow-up tests.

**Figure 11:**
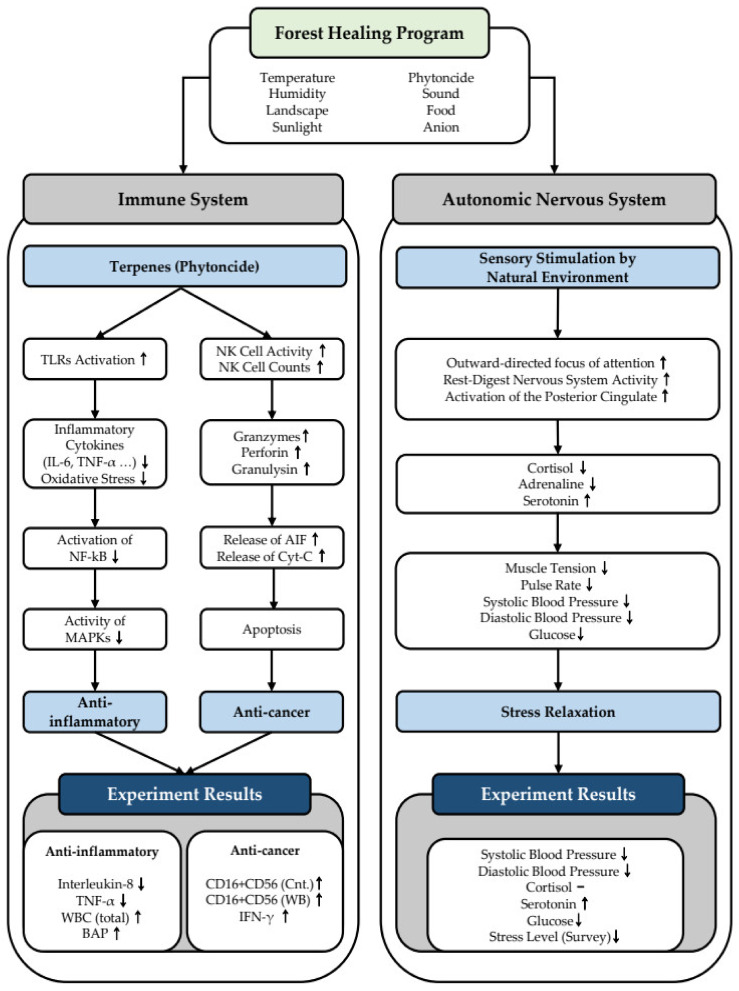
Mechanism of the forest healing program. TLRs: Toll-like receptors; NF-kB: nuclear factor kappa-light-chain-enhancer of activated B cells; AIF: apoptosis-inducing factor; Cyt-C: cytochrome complex.

Categorizing the results derived from this study according to health effects, the first health promotion effect that can be expected through forest healing programs is immunity enhancement (Figure 11). Among the various factors involved in forest healing, phytoncide is known to have excellent anti-bacterial and anti-inflammatory effects, as it activates Toll-like receptors in the body, reducing inflammatory cytokines, such as IL-6 and TNF-α, as well as oxidative stress. [74]. Additionally, it has been shown to inhibit the nuclear factor kappa-enhancer of activated B cells (NF-kB), which are essential for viral protein production, and inhibit the activation of mitogen-activated protein kinases. Prior studies have demonstrated the anti-inflammatory effects of phytoncide in the body [74]. This study also revealed the anti-inflammatory effects of forest healing in that both IL-8 and TNF-α, which are associated with inflammation in vivo, significantly decreased after the forest healing program. Meanwhile, phytoncide has significant effects on activating the natural killer (NK) cells in the body and increasing the number of NK cells. NK cells play an important role in eliminating cancer cells, and NK cell activation and an increase in their number facilitate anticancer activity [75]. In particular, as NK activity significantly increases in cells exposed to phytoncide for more than 144 h, it is important to reflect the long-term exposure to phytoncide during the establishment and planning of future forest healing programs [75]. Looking at the mechanism of chemotherapy through phytoncide, phytoncide promotes NK cell activation and increases the number of NK cells, which increases granzymes, perforin, and granulysin, which, in turn, cause necrosis of the target cells. It then increases cytochrome-C and an apoptosis inducing factor, which induces the apoptosis of cancer cells, resulting in anticancer activity in the body [75]. Therefore, the significant changes in CD16+CD56 (count), CD16+CD56 (WB), and IFN-γ through the forest healing program observed in this study demonstrate that forest healing programs have a positive effect on the activity of NK cells, and effectively enhance anticancer activity and immunity.

The second expected health-promoting effect of forest healing programs demonstrated in this study is related to stress recovery (Figure 11). Theories of natural preferences posit that humans instinctively prefer nature, as explained through the biophilia and savannah hypotheses. [76] Furthermore, attention restoration theory interprets that humans who are constantly exposed to artificial environments instinctively visit nature [76,77]. In addition, psycho-evolutionary theory suggests that exposure to the natural environment leads to positive psychological changes and reduces stress. [76]. Meanwhile, forest healing shows excellent preventive medical effects by relieving physical fatigue and promoting immune function recovery through a natural recovery environment that stimulates the five senses [78]. Several prior studies have argued for the stress reduction effect of forest healing, and recently, attempts have been made to scientifically explain the effects and mechanisms of stress reduction by nature based on neuroimaging using functional magnetic resonance imaging. Sensory stimulation by looking at natural scenery or listening to natural sounds affects the autonomic nervous system and reduces stress [79,80]. Sensory stimulation through nature promotes an outward-directed focus of attention and rest-digest nervous system activity, and activates the posterior cingulate, a part of the brain that responds to emotions. These changes in the brain reduce stress-related hormones, such as cortisol and adrenaline, and increase serotonin levels. These hormonal changes cause physical stress relief, including reduced muscle tension, blood pressure, and pulse rate. This study found evidence to support these claims. Significant positive changes were observed in cardiovascular-related indicators, with no significant changes in cortisol levels, but significant increases in serotonin levels, which helps relieve stress. In addition, the study found that participants’ stress continued to decrease after the forest healing program.

It is important to note that this study has several limitations. The main limitation of this study is that there is no control group that includes participants who have not participated in the forest healing program; thus, the results of this study may need to be analyzed in conjunction with the results of subsequent studies that will involve control groups. This study conducted experiments on adolescents living relatively homogeneous lives, making it difficult to generalize the health promotion effects of the forest healing program. In addition, the analysis results may not have been accurate due to the effects of the growth period, and the lack of control over other factors, such as smoking and eating habits, remains a limitation. Therefore, future studies need to investigate and account for the life patterns and drinking and smoking habits of the participants that could negatively affect the results. Further, it is believed that fear, triggered by clinical assessments, may have affected the result outcomes of the participants. 

While it is most desirable to measure changes in health conditions across the entire sample at five points during the study, including pre-, post-, and follow-up (one week, two weeks, and four weeks later) tests, the study design used only three time points for assessment considering that the participants were adolescents, and because it is difficult to track down all the participants for five examinations. Therefore, the participants were divided into three groups for more efficient clinical assessment. After testing for the homogeneity of the participants, the results of all the participants were presented in pre-, post-, and follow-up tests, and the follow-up test results were sub-divided into those conducted one, two, and four weeks after the program. Data collection was attempted at the same time of the day, but voluntary participation made it difficult to control the exact time of assessment. It is considered that significant results were difficult to observe for indicators such as cortisol and those related to circadian rhythm, as difficulty in controlling the timing of sample collection and fear of testing are likely to affect biological results. 

In the case of saliva measurement, careful on-field examination is necessary because it was observed that there were too many missing values for analysis. On-site sampling of saliva is relatively easy and convenient, but researchers should be careful that analysis may be difficult. However, if the right measurement can be obtained at the site in the future, it will be beneficial to analyze the forest healing effects with relative ease. 

This study is significant, as it assessed the health indicators of the effects of the forest healing program at three time points rather than only conducting a short-term analysis through pre- and post-treatment comparisons, such as that seen in previous studies. However, the preliminary examination was conducted two weeks before the program, which may not have accurately measured indicators, the results of which change rapidly in a short period of time, such as cortisol. Therefore, subsequent studies in which pre- and post-measurements can be performed within one day of the program should be conducted to increase the accuracy of the results.

In addition, this forest healing program included an indoor program; therefore, during the three-day and two-night program, the actual time spent in the forest was less than 10 h. However, based on the literature that shows that the short forest walking programs of 15–40 min had physical and psychological health promotion effects, the healing effect of the forest healing program conducted in this study is expected to be sufficient; nevertheless, increasing the proportion of forest activities in future forest healing programs is expected to have a more pronounced effect [16]. 

Furthermore, as the program was conducted for healthy individuals, and not patients, most indicators showed changes within the normal range, making it difficult to observe the dramatic health promotion effect of forest healing. However, after the forest healing program, the number of outliers (outcomes outside the normal range) was significantly reduced, and significant health promotion effects were observed in several indicators, including blood pressure. 

Although forest healing had a positive impact on various health-related indicators, including the autonomic nervous system, it was also found that the nature of forest healing led to various allergic reactions in participants. Higher levels of inflammation were observed in several participants due to allergic sensitization reactions, suggesting that forest healing programs may have a negative effect on those with allergies as shown in Appendix D. Analysis of IL-8, an allergen-related indicator, showed an allergic sensitization rate of 54.93% in the group that showed increased levels of IL-8 after the forest healing program, which was much higher than 41.18%, which was the allergic sensitization rate of the group that showed decreased levels of IL-8 after the forest healing program. For both the groups with increased and decreased values, the *t*-test showed that the significance levels were below 0.001, indicating significant changes. However, no clear evidence has been found that forest healing programs cause allergic reactions and increase inflammation levels, and it is necessary to proceed with the program in consideration of this in the future. It is expected that when conducting a forest healing program, a process of selecting the appropriate place, time, and type of program will be needed for each participant.

Based on these limitations, future studies should attempt to build forest healing big data infrastructure and systems through sampling using non-invasive methods, investigating other influential factors affecting test results, and linking sensing and wearable technologies. This study is significant, as it involved a relatively large number of participants compared to small samples in the studies conducted previously, and also as it conducted a pilot study to quantify forest healing effects using various physiological indicators, such as urine and blood, as well as psychological indicators. Therefore, to establish an evidence-based forest healing program, time-series changes could be measured with varying frequency and duration, and the persistence and continuity as well as the frequency and cycle of forest healing effects could be investigated for indicators for which the effects have continued after the cessation of the forest healing program, or the appearance of effects has been relatively delayed.

## 5. Conclusions

The study showed that the three-day and two-night forest healing program had a generally positive effect on the physiological and psychological health of the participants. Of the 87 health-related indicators, significant impacts were observed for 46 indicators, such as cardiovascular, immune function, and mental health. In this study, the effectiveness of forest healing was assessed from various perspectives by using non-universal clinical indicators. Furthermore, beyond the comparison of pre- and post-treatment, changes were observed one week, two weeks, and four weeks after the program. Therefore, this study provides a basis for selecting appropriate indicators when developing a long-term follow-up survey system in the future and is significant as a framework for evaluating forest healing effects and promoting related policies.

## Figures and Tables

**Table 1:** Demographic characteristics of the participants.

Indicators		Total	Male	Female
		Mean ± SD/n (%)	Mean ± SD/n (%)	Mean ± SD/n (%)
Gender		88 (100)	49 (55.7)	39 (44.3)
Age (years)	15 and below	46 (52.3)	27 (55.1)	19 (48.7)
	16 and above	42 (47.7)	22 (44.9)	20 (51.3)
Region	Gimcheon	30 (34.0)	18 (36.7)	12 (30.8)
	Daejeon	29 (33.0)	18 (36.7)	11 (28.2)
	Incheon	29 (33.0)	13 (26.6)	16 (41.0)
Forest Healing Experience	None	60 (68.2)	35 (71.4)	25 (64.1)
	Once	26 (29.5)	12 (24.5)	14 (35.9)
	Twice	2 (2.3)	2 (4.1)	0 (0.0)
**Health Examination Indicators**		**Mean±SD**	**Mean±SD**	Mean±SD
Health Examination	Height(cm)	165.14 ± 7.64	169.10 ± 5.99	160.08 ± 6.49
	Weight(kg)	58.54 ± 10.44	59.95 ± 9.38	56.76 ± 11.52
	BMI	21.44 ± 3.45	20.91 ± 2.76	22.12 ± 4.12
	Total Body Water	33.31 ± 6.07	36.73 ± 4.57	28.94 ± 4.82
	Protein	8.90 ± 1.81	9.90 ± 1.28	7.63 ± 1.57
	Mineral	3.20 ± 0.54	3.45 ± 0.45	2.87 ± 0.47
	Body Fat Mass	13.08 ± 7.28	9.86 ± 5.47	17.19 ± 7.29
	Soft Lean Mass	42.68 ± 8.07	47.23 ± 5.91	36.89 ± 6.64
	Fat Free Mass	45.35 ± 8.47	50.08 ± 6.28	39.32 ± 6.94

**Table 2:** NDVI analysis of participants’ living areas.

Region	Classification	Mean	SD	Median
Gimcheon	Living Area (500 m)	0.2646	0.1043	0.2577
	Walking Distance Area (1000 m)	0.2736	0.1102	0.2724
Daejeon	Living Area (500 m)	0.0560	0.0670	0.2699
	Walking Distance Area (1000 m)	0.2436	0.0771	0.2435
Incheon	Living Area (500 m)	0.2330	0.1124	0.2478
	Walking Distance Area (1000 m)	0.2077	0.1137	0.1645

**Table 3:** Site forest vegetation.

% of Forest Area	91.24%
Crown Density	79.20%
NDVI	0.392
Vegetation Community	
*Larix kaempferi*	69.37
*Quercus aliena*	16.29
Deciduous broad-leaved	3.23
*Pinus densiflora*	3.05
*Abies holophylla*	1.74
*Betula platyphylla*	1.73
*Pinus koraiensis*	1.63
Quercus-Pinus mixed	1.46

The natural environment around the study site was analyzed using GIS. The data were extracted from remote sensing, such as Landsat 8, forest cover map, and land cover map. The analysis covers 500 m buffered from Heongseong SoopChewon.

**Table 4:** Forest healing program schedule.

Day	Time	Program
Day 1	14:00–15:00	Orientation and safety education
	16:00–17:30	Forest trail walking and do-it-yourself glass bottle crafts
	19:00–20:30	Building structures with wood blocks in forests
	20:30	Night forest walking
Day 2	9:30–12:00	Group forest trekking with hidden missions
	14:00–15:30	Making a wooden clock
	15:30–16:00	
	16:00–17:00	Lecture on encouraging independence
	19:00–20:30	Listening to music in forests
	20:30	Night forest walking
Day 3	9:30–11:30	Lecture from seniors Post-treatment measurement

**Table 5:** Measurement factors.

	Methods	Indicators
Physiological Indicator	Physical Examination	Systolic BP; Diastolic BP
		eNO
		LnTP; LnLF; LnHF; LnLF/HF; RMSDD; pNN50
	Complete Blood Count	RBC
		Platelet
		Total WBC; Lymphocytes; Monocytes; Eosinophils; Basophils
	Biochemical Examination	Cortisol; Serotonin
		d-ROMs
		BAP (Biological Antioxidant Potential)
		25-(OH) Vitamin D; 1,25-(OH)3 Vitamin D
		SGOT(AST); SGPT(ALT)
		Total protein; Albumin; Bilirubin; BUN (Blood Urea Nitrogen); Creatinine; Glucose
	Immuno- serological Examination	IL-4 HS Multiplex; IL-8 Multiplex
		TNF-α HS Multiplex
		IFN-γ HS Multiplex
		CD16+CD56 (count); CD16+CD56 (WB)
		IgA
	Saliva test	Secretory IgA (Saliva)
		Cortisol (Saliva)
	Urine test	8-OHdG
Psychological Indicator	Self-report Survey	Resilience
		Interpersonal competency
		Self-esteem
		Stress response
		Vigor

## Data Availability

The data presented in this study are available in the article and appendices.

## Appendix A

**Table A1:** Result Table of Descriptive Statistics, One-way RM ANOVA, and Paired *t*-Test.

Classification	Indicators	Gender	Pre-	Post-	Follow-Up	*p*	Daily	Post-	*p*
			M(SD)	M(SD)	M(SD)		M(SD)	M(SD)	
Blood Pressure and Autonomic Nervous System	Systolic BP	Total	123.58(15.48)	117.93(12.77)	118.52(12.67)	***	121.05(14.33)	117.93(12.77)	**
		Male	127.49(15.58)	120.04(13.66)	122.78(13.45)	**	125.27(14.73)	120.04(13.66)	**
		Female	118.67(14.05)	115.28(11.17)	113.18(9.29)	*	115.76(11.94)	115.28(11.17)	
		Group 1	127.97(15.27)	119.87(12.68)	116.16(11.09)	***	121.92(14.73)	119.87(12.68)	
		Group 2	129.00(15.26)	116.45(14.30)	125.21(12.93)	***	127.10(14.15)	116.45(14.30)	***
		Group 3	113.62(10.41)	117.48(11.14)	114.59(11.52)		114.10(10.90)	117.48(11.14)	*
	Diastolic BP	Total	76.33(11.51)	73.73(8.94)	71.66(9.75)	***	73.99(10.89)	73.73(8.94)	
		Male	75.69(10.69)	73.16(9.98)	69.61(10.06)	***	72.74(10.82)	73.16(9.98)	
		Female	77.13(12.56)	74.44(7.51)	74.23(8.82)		75.56(10.85)	74.44(7.51)	
		Group 1	74.84(13.72)	72.87(7.64)	70.06(11.61)		72.23(12.83)	72.87(7.64)	
		Group 2	79.86(10.12)	72.45(11.28)	74.10(8.99)	***	76.98(9.93)	72.45(11.28)	***
		Group 3	74.10(9.45)	75.55(7.81)	70.41(7.95)	**	72.83(9.03)	75.55(7.81)	*
	RMSSD	Total	57.77(36.36)	43.31(27.16)	43.07(23.28)	***	50.42(31.32)	43.31(27.16)	**
		Male	68.00(41.18)	46.10(28.08)	47.59(25.44)	***	57.86(35.54)	46.10(28.08)	**
		Female	44.92(24.09)	39.79(25.90)	37.38(19.07)		41.08(21.90)	39.79(25.90)	
		Group 1	60.10(31.50)	34.93(20.42)	39.83(20.70)	***	49.97(28.33)	34.93(20.42)	***
		Group 2	65.69(45.66)	51.90(33.04)	49.03(30.53)		57.36(39.40)	51.90(33.04)	
		Group 3	47.45(28.53)	43.38(24.85)	40.45(15.91)		43.95(23.16)	43.38(24.85)	
	LnTP	Total	7.13(0.54)	7.00(0.51)	6.97(0.44)	*	7.05(0.50)	7.00(0.51)	
		Male	7.33(0.56)	7.10(0.48)	7.07(0.42)	***	7.20(0.51)	7.10(0.48)	*
		Female	6.87(0.41)	6.87(0.53)	6.84(0.43)		6.85(0.41)	6.87(0.53)	
		Group 1	7.23(0.57)	6.81(0.37)	6.89(0.47)	***	7.06(0.55)	6.81(0.37)	***
		Group 2	7.20(0.56)	7.18(0.67)	7.05(0.44)		7.13(0.51)	7.18(0.67)	
		Group 3	6.93(0.45)	7.01(0.39)	6.98(0.39)		6.96(0.42)	7.01(0.39)	
	LnLF	Total	5.30(1.13)	5.22(1.05)	5.01(1.13)		5.15(1.14)	5.22(1.05)	
		Male	5.69(1.04)	5.42(1.01)	5.27(1.05)	*	5.48(1.06)	5.42(1.01)	
		Female	4.82(1.07)	4.97(1.05)	4.68(1.14)		4.74(1.11)	4.97(1.05)	
		Group 1	5.35(1.09)	4.78(0.82)	4.92(1.15)	*	5.14(1.13)	4.78(0.82)	*
		Group 2	5.52(1.19)	5.53(1.33)	5.11(1.18)		5.31(1.19)	5.53(1.33)	
		Group 3	5.04(1.10)	5.38(0.77)	4.99(1.09)		5.01(1.09)	5.38(0.77)	**
	LnHF	Total	5.12(1.15)	4.72(0.98)	4.76(1.02)	***	4.94(1.10)	4.72(0.98)	**
		Male	5.45(1.04)	4.83(0.97)	4.95(1.00)	***	5.19(1.05)	4.83(0.97)	***
		Female	4.71(1.18)	4.57(0.98)	4.52(1.01)		4.63(1.09)	4.57(0.98)	
		Group 1	5.41(1.04)	4.47(0.81)	4.60(1.04)	***	5.01(1.11)	4.47(0.81)	***
		Group 2	5.14(1.23)	4.96(1.18)	4.86(1.16)		5.00(1.19)	4.96(1.18)	
		Group 3	4.80(1.15)	4.73(0.89)	4.82(0.86)		4.81(1.00)	4.73(0.89)	
	LnLF/LnHF	Total	1.06(0.19)	1.13(0.20)	1.06(0.19)	**	1.06(0.19)	1.13(0.20)	***
		Male	1.06(0.18)	1.14(0.20)	1.08(0.19)	*	1.07(0.18)	1.14(0.20)	**
		Female	1.05(0.20)	1.11(0.20)	1.04(0.20)		1.04(0.20)	1.11(0.20)	**
		Group 1	1.00(0.17)	1.09(0.22)	1.08(0.14)		1.04(0.16)	1.09(0.22)	
		Group 2	1.09(0.20)	1.13(0.18)	1.07(0.23)		1.08(0.21)	1.13(0.18)	
		Group 3	1.07(0.19)	1.16(0.18)	1.05(0.21)	*	1.06(0.20)	1.16(0.18)	***
	pNN50	Total	67.10(15.87)	62.84(16.84)	64.27(17.08)		65.69(16.50)	62.84(16.84)	*
		Male	71.12(11.61)	62.94(16.38)	64.18(17.16)	***	67.74(15.05)	62.94(16.38)	**
		Female	62.05(18.96)	62.72(17.60)	64.38(17.19)		63.10(17.93)	62.72(17.60)	
		Group 1	71.43(18.08)	59.33(17.46)	59.00(20.49)	**	65.22(20.16)	59.33(17.46)	*
		Group 2	65.55(16.32)	67.03(16.92)	64.24(16.36)		64.90(16.21)	67.03(16.92)	
		Group 3	64.17(12.10)	62.28(15.71)	69.76(11.95)	*	66.97(12.25)	62.28(15.71)	*
Immune Function and Inflammation	CD16+CD56 (Count)	Total	317.12(155.53)	279.06(139.67)	320.12(180.46)	*	318.62(167.98)	279.06(139.67)	***
		Male	294.24(129.07)	272.09(146.09)	314.72(198.45)		308.81(163.74)	272.09(146.09)	*
		Female	345.85(181.14)	287.81(132.51)	326.92(157.24)		330.94(173.42)	287.81(132.51)	**
		Group 1	301.49(152.92)	318.63(159.84)	364.00(172.09)	**	332.74(164.45)	318.63(159.84)	
		Group 2	284.50(155.22)	250.83(108.97)	268.57(204.46)		276.53(180.10)	250.83(108.97)	
		Group 3	365.90(151.71)	266.35(140.01)	326.28(154.44)	**	346.09(153.04)	266.35(140.01)	***
	CD16+CD56 (WB)	Total	14.13(6.06)	12.49(5.22)	12.49(5.87)	**	13.31(6.00)	12.49(5.22)	*
		Male	13.61(5.23)	12.35(5.63)	12.56(6.82)		13.28(5.94)	12.35(5.63)	
		Female	14.79(6.97)	12.67(4.70)	12.40(4.48)	*	13.36(6.12)	12.67(4.70)	
		Group 1	13.53(6.24)	14.66(6.35)	15.01(5.97)		14.27(6.10)	14.66(6.35)	
		Group 2	15.27(7.36)	11.48(4.26)	9.96(5.84)	***	12.61(7.11)	11.48(4.26)	
		Group 3	13.62(4.18)	11.25(4.12)	12.42(4.73)	*	13.02(4.47)	11.25(4.12)	**
	IFN-γ HS M_Multiplex	Total	31.81(15.90)	32.52(21.35)	34.86(18.51)	*	33.33(17.27)	32.52(21.35)	
		Male	29.30(12.48)	27.08(10.51)	30.51(14.12)	*	29.67(13.46)	27.08(10.51)	***
		Female	34.96(19.08)	39.34(28.60)	40.32(21.85)		37.94(20.28)	39.34(28.60)	
		Group 1	28.44(11.72)	29.47(13.63)	36.11(15.70)	***	32.28(14.27)	29.47(13.63)	**
		Group 2	32.47(12.38)	26.74(9.21)	33.75(16.38)	**	33.11(14.40)	26.74(9.21)	***
		Group 3	34.64(21.67)	41.45(31.81)	34.67(23.20)	*	34.66(22.25)	41.45(31.81)	**
	TNF-α HS M_Multiplex	Total	16.50(3.80)	14.46(3.75)	14.16(3.73)	***	15.33(3.93)	14.46(3.75)	**
		Male	17.40(4.31)	14.96(4.17)	14.55(4.24)	***	16.08(4.46)	14.96(4.17)	**
		Female	15.36(2.68)	13.84(3.09)	13.67(2.96)	**	14.38(2.90)	13.84(3.09)	
		Group 1	16.11(2.99)	14.66(3.52)	15.50(3.56)	*	15.80(3.28)	14.66(3.52)	**
		Group 2	17.66(3.81)	12.84(3.36)	13.22(2.57)	***	15.44(3.92)	12.84(3.36)	***
		Group 3	15.74(4.34)	15.88(3.83)	13.71(4.52)	**	14.73(4.51)	15.88(3.83)	*
	IL-4 HS M_Multiplex	Total	84.64(58.53)	81.81(60.05)	78.72(58.69)		81.68(58.52)	81.81(60.05)	
		Male	80.06(50.30)	74.46(44.46)	74.56(48.07)		76.71(49.46)	74.46(44.46)	
		Female	90.40(67.71)	91.04(74.84)	83.95(70.12)		87.93(68.04)	91.04(74.84)	
		Group 1	69.28(40.10)	70.40(48.18)	83.04(44.94)	**	76.16(42.80)	70.40(48.18)	
		Group 2	100.70(67.04)	77.72(48.14)	88.74(60.56)	***	94.72(63.60)	77.72(48.14)	***
		Group 3	84.48(62.96)	97.70(77.87)	64.24(67.84)	***	74.36(65.67)	97.70(77.87)	***
	IL-8 M_Multiplex	Total	20.52(24.31)	73.70(81.42)	20.94(27.62)	***	20.73(25.95)	73.70(81.42)	***
		Male	20.71(26.97)	80.70(77.90)	22.80(34.68)	***	21.63(30.97)	80.70(77.90)	***
		Female	20.28(20.84)	64.91(85.85)	18.60(14.75)	***	19.60(17.87)	64.91(85.85)	***
		Group 1	12.12(9.71)	118.79(107.18)	17.13(11.13)	***	14.73(10.68)	118.79(107.18)	***
		Group 2	16.81(11.16)	78.00(56.71)	13.40(12.56)	***	15.11(11.90)	78.00(56.71)	***
		Group 3	32.91(36.96)	22.76(24.52)	32.21(43.37)	*	32.56(39.94)	22.76(24.52)	**
	IgA(S)	Total	183.66(65.05)	183.11(62.12)	179.10(59.99)		181.38(62.43)	183.11(62.12)	
		Male	185.88(52.14)	186.89(51.90)	181.27(49.37)	**	183.04(50.69)	186.89(51.90)	*
		Female	180.87(78.99)	178.37(73.42)	176.38(71.75)		179.29(74.92)	178.37(73.42)	
		Group 1	178.43(54.04)	180.78(52.18)	178.06(52.73)		178.25(52.94)	180.78(52.18)	*
		Group 2	182.48(57.38)	181.32(54.42)	175.86(53.26)	**	179.17(54.97)	181.32(54.42)	
		Group 3	190.25(82.18)	187.31(78.61)	183.42(73.72)		186.84(77.46)	187.31(78.61)	
	eNO	Total	15.93(18.53)	17.33(19.62)	16.10(16.12)		16.02(17.32)	17.33(19.62)	*
		Male	18.41(22.09)	20.39(24.06)	19.10(19.77)		18.40(20.88)	20.39(24.06)	
		Female	12.82(12.33)	13.49(11.09)	12.41(8.70)		13.03(10.78)	13.49(11.09)	
		Group 1	13.42(8.66)	16.23(12.12)	13.52(8.23)	*	13.05(8.15)	16.23(12.12)	*
		Group 2	22.21(29.68)	22.43(29.44)	18.68(22.15)	*	20.23(25.58)	22.43(29.44)	*
		Group 3	13.17(9.48)	15.14(14.92)	16.59(15.77)		14.88(13.01)	15.14(14.92)	
	Basophil	Total	0.90(0.39)	0.71(0.29)	0.76(0.32)	***	0.83(0.37)	0.71(0.29)	***
		Male	0.95(0.39)	0.76(0.25)	0.78(0.36)	***	0.88(0.38)	0.76(0.25)	*
		Female	0.85(0.39)	0.64(0.32)	0.74(0.28)	**	0.77(0.34)	0.64(0.32)	***
		Group 1	0.74(0.33)	0.74(0.33)	0.75(0.35)		0.74(0.34)	0.74(0.33)	
		Group 2	1.22(0.37)	0.69(0.25)	0.83(0.32)	***	1.03(0.39)	0.69(0.25)	***
		Group 3	0.76(0.27)	0.69(0.29)	0.70(0.30)		0.73(0.28)	0.69(0.29)	
	Lymphocyte	Total	36.14(9.08)	36.14(8.80)	35.75(8.13)		35.95(8.60)	36.14(8.80)	
		Male	36.83(9.35)	37.15(8.10)	36.64(8.52)		37.03(8.79)	37.15(8.10)	
		Female	35.28(8.77)	34.87(9.57)	34.63(7.58)		34.59(8.20)	34.87(9.57)	
		Group 1	32.66(8.34)	37.59(8.77)	35.82(7.12)	**	34.24(7.85)	37.59(8.77)	**
		Group 2	39.41(10.74)	36.59(9.01)	35.98(9.58)	*	37.69(10.24)	36.59(9.01)	
		Group 3	36.48(6.68)	34.19(8.58)	35.44(7.81)		35.96(7.22)	34.19(8.58)	
	Eosinophil	Total	2.34(1.84)	3.03(2.27)	2.52(2.61)	**	2.43(2.25)	3.03(2.27)	***
		Male	2.53(1.92)	3.21(2.37)	2.79(3.25)		2.67(2.66)	3.21(2.37)	*
		Female	2.10(1.73)	2.79(2.14)	2.17(1.41)	**	2.12(1.56)	2.79(2.14)	***
		Group 1	1.88(1.74)	3.11(2.52)	2.48(1.89)	***	2.18(1.82)	3.11(2.52)	***
		Group 2	2.90(2.17)	3.14(2.13)	2.30(1.55)	**	2.60(1.89)	3.14(2.13)	**
		Group 3	2.27(1.46)	2.82(2.19)	2.77(3.86)		2.52(2.90)	2.82(2.19)	
	Monocyte	Total	8.45(2.14)	8.22(1.82)	7.57(1.63)	***	8.01(1.95)	8.22(1.82)	
		Male	8.61(2.07)	8.79(1.83)	7.88(1.70)	**	8.32(1.92)	8.79(1.83)	*
		Female	8.25(2.23)	7.51(1.55)	7.17(1.47)	***	7.62(1.92)	7.51(1.55)	
		Group 1	7.81(1.82)	8.32(1.88)	7.26(1.43)	*	7.54(1.65)	8.32(1.88)	**
		Group 2	9.61(2.28)	8.12(1.82)	7.64(1.36)	***	8.63(2.11)	8.12(1.82)	
		Group 3	7.96(1.86)	8.22(1.82)	7.81(2.03)		7.88(1.93)	8.22(1.82)	
Oxidative Stress and Antioxidant	8-OHdG	Total	12.31(6.96)	13.14(5.89)	11.92(5.93)		12.11(6.45)	13.14(5.89)	
		Male	12.75(6.37)	12.98(5.95)	11.70(6.16)		12.13(6.25)	12.98(5.95)	
		Female	11.75(7.69)	13.35(5.89)	11.90(5.99)		12.09(6.73)	13.35(5.89)	*
		Group 1	11.73(5.95)	14.69(6.59)	12.47(6.52)		12.10(6.20)	14.69(6.59)	*
		Group 2	11.60(6.78)	12.95(6.56)	11.43(5.87)		11.52(6.29)	12.95(6.56)	
		Group 3	13.61(8.08)	11.73(3.91)	11.42(5.91)		12.72(6.90)	11.73(3.91)	
	d-ROMs	Total	311.01(64.73)	312.82(64.74)	314.88(64.92)		312.94(64.67)	312.82(64.74)	
		Male	299.88(57.26)	297.33(61.65)	299.24(58.55)		296.56(54.35)	297.33(61.65)	
		Female	325.00(71.34)	332.28(64.01)	334.51(67.89)		333.53(70.80)	332.28(64.01)	
		Group 1	314.60(62.27)	320.30(66.39)	319.83(65.82)		317.22(63.58)	320.30(66.39)	
		Group 2	314.24(72.53)	314.90(68.87)	307.45(73.36)		310.84(72.38)	314.90(68.87)	
		Group 3	304.07(60.55)	303.00(59.59)	317.17(56.02)	*	310.62(58.19)	303.00(59.59)	
	BAP	Total	2133.88(156.87)	2182.43(162.15)	1944.57(229.48)	***	2039.22(217.77)	2182.43(162.15)	***
		Male	2180.98(146.73)	2227.69(158.84)	1973.63(231.93)	***	2078.82(220.65)	2227.69(158.84)	***
		Female	2074.69(150.63)	2125.56(149.55)	1908.05(223.96)	***	1989.47(204.82)	2125.56(149.55)	***
		Group 1	2138.30(140.26)	2171.33(166.45)	1840.37(131.38)	***	1989.33(201.80)	2171.33(166.45)	***
		Group 2	2130.21(173.30)	2176.17(148.53)	1850.76(204.81)	***	1990.48(235.00)	2176.17(148.53)	***
		Group 3	2132.97(161.29)	2200.17(174.47)	2146.17(202.37)		2139.57(181.50)	2200.17(174.47)	**
Stress (Hormone)	Cortisol (CIA)	Total	6.43(2.47)	6.62(2.64)	6.59(2.91)		6.51(2.69)	6.62(2.64)	
		Male	6.41(2.51)	6.62(2.81)	6.59(3.38)		6.50(2.99)	6.62(2.81)	
		Female	6.33(2.60)	6.61(2.44)	6.59(2.21)		6.54(2.29)	6.61(2.44)	
		Group 1	5.81(2.78)	6.23(2.54)	5.55(1.96)		5.79(2.27)	6.23(2.54)	
		Group 2	6.74(2.26)	6.34(2.78)	7.32(3.20)		7.03(2.76)	6.34(2.78)	
		Group 3	6.55(2.61)	7.30(2.55)	6.94(3.19)		6.74(2.90)	7.30(2.55)	
	Cortisol (Saliva)	Total	0.10(0.08)	0.10(0.06)	0.11(0.07)	-	0.11(0.07)	0.10(0.06)	
		Male	0.09(0.07)	0.10(0.05)	0.11(0.07)	-	0.10(0.07)	0.10(0.05)	
		Female	0.11(0.09)	0.09(0.06)	0.11(0.06)	-	0.11(0.07)	0.09(0.06)	
		Group 1	0.09(0.09)	0.10(0.03)	0.10(0.05)	-	0.09(0.06)	0.10(0.03)	
		Group 2	0.09(0.05)	0.07(0.04)	0.15(0.09)	-	0.12(0.08)	0.07(0.04)	**
		Group 3	0.10(0.09)	0.11(0.07)	0.10(0.05)	-	0.10(0.07)	0.11(0.07)	
	Serotonin	Total	145.97(48.27)	150.45(62.59)	168.88(62.81)	***	157.42(57.02)	150.45(62.59)	*
		Male	145.72(45.27)	150.61(62.41)	175.00(67.87)	***	161.04(58.94)	150.61(62.41)	*
		Female	146.28(52.39)	150.25(63.63)	161.18(55.70)		152.87(54.55)	150.25(63.63)	
		Group 1	152.26(44.52)	138.55(41.34)	177.58(50.76)	***	164.92(49.03)	138.55(41.34)	***
		Group 2	155.76(52.38)	186.54(79.57)	192.77(74.88)	***	174.26(66.71)	186.54(79.57)	
		Group 3	129.68(45.01)	126.67(44.53)	135.98(46.78)	*	132.83(45.61)	126.67(44.53)	
Health Screening Parameters	25-(OH) Vitamin D	Total	20.03(6.10)	21.11(5.60)	21.72(5.38)	***	20.88(5.79)	21.11(5.60)	
		Male	21.86(6.03)	22.98(5.51)	23.48(4.87)	***	22.74(5.39)	22.98(5.51)	
		Female	17.74(5.43)	18.76(4.82)	19.52(5.23)	**	18.54(5.45)	18.76(4.82)	
		Group 1	21.89(7.08)	21.31(6.59)	21.72(6.53)		21.81(6.75)	21.31(6.59)	*
		Group 2	19.62(4.47)	21.15(5.02)	21.63(4.59)	***	20.63(4.60)	21.15(5.02)	
		Group 3	18.52(6.11)	20.86(5.21)	21.81(4.97)	***	20.17(5.76)	20.86(5.21)	
	1, 25-(OH) 3 Vitamin D	Total	27.60(15.72)	13.81(15.64)	32.06(19.26)	***	29.83(17.67)	13.81(15.64)	***
		Male	29.64(15.68)	16.74(18.36)	33.38(19.46)	***	31.63(17.54)	16.74(18.36)	***
		Female	25.03(15.59)	10.13(10.45)	30.42(19.13)	***	27.57(17.69)	10.13(10.45)	***
		Group 1	29.18(16.63)	8.53(7.23)	23.37(16.7)	***	26.28(16.48)	8.53(7.23)	***
		Group 2	27.86(14.34)	23.16(22.31)	22.97(12.12)		25.41(13.39)	23.16(22.31)	
		Group 3	25.69(16.41)	9.93(8.49)	50.16(15.25)	***	37.93(19.97)	9.93(8.49)	***
	Glucose	Total	86.40(11.97)	83.77(9.75)	96.59(13.74)	***	91.49(13.83)	83.77(9.75)	***
		Male	86.04(11.33)	85.24(9.88)	97.67(13.96)	***	91.47(13.82)	85.24(9.88)	***
		Female	86.85(12.86)	81.92(9.38)	95.23(13.51)	***	91.53(13.92)	81.92(9.38)	***
		Group 1	83.77(12.40)	80.80(9.48)	97.63(13.27)	***	90.70(14.53)	80.80(9.48)	***
		Group 2	88.62(9.33)	87.76(8.96)	98.93(10.60)	***	93.78(11.18)	87.76(8.96)	**
		Group 3	86.90(13.63)	82.86(9.76)	93.17(16.51)	*	90.03(15.34)	82.86(9.76)	**
	BUN	Total	12.17(3.06)	11.03(2.68)	12.53(2.60)	***	12.35(2.84)	11.03(2.68)	***
		Male	12.65(2.88)	11.29(2.18)	12.92(2.52)	***	12.82(2.68)	11.29(2.18)	***
		Female	11.56(3.21)	10.72(3.19)	12.05(2.67)	*	11.77(2.94)	10.72(3.19)	***
		Group 1	11.23(2.86)	9.97(2.85)	13.27(3.05)	***	12.25(3.11)	9.97(2.85)	***
		Group 2	11.90(3.02)	10.93(2.51)	11.62(2.51)		11.76(2.75)	10.93(2.51)	*
		Group 3	13.41(2.98)	12.24(2.20)	12.69(1.91)		13.05(2.51)	12.24(2.20)	*
	Creatinine	Total	0.76(0.15)	0.73(0.14)	0.77(0.14)	***	0.77(0.15)	0.73(0.14)	***
		Male	0.82(0.15)	0.78(0.14)	0.82(0.14)	**	0.83(0.14)	0.78(0.14)	***
		Female	0.69(0.13)	0.66(0.10)	0.70(0.12)	*	0.69(0.11)	0.66(0.10)	***
		Group 1	0.73(0.16)	0.73(0.15)	0.76(0.16)		0.75(0.16)	0.73(0.15)	
		Group 2	0.78(0.15)	0.75(0.12)	0.79(0.13)	*	0.79(0.14)	0.75(0.12)	**
		Group 3	0.78(0.15)	0.70(0.13)	0.76(0.14)	***	0.77(0.15)	0.70(0.13)	***
	Bilirubin	Total	0.46(0.24)	0.47(0.24)	0.49(0.29)		0.47(0.27)	0.47(0.24)	
		Male	0.51(0.23)	0.51(0.25)	0.53(0.29)		0.52(0.26)	0.51(0.25)	
		Female	0.39(0.23)	0.43(0.22)	0.43(0.29)		0.41(0.26)	0.43(0.22)	
		Group 1	0.47(0.26)	0.51(0.25)	0.52(0.28)		0.49(0.27)	0.51(0.25)	
		Group 2	0.54(0.24)	0.48(0.20)	0.51(0.28)		0.52(0.26)	0.48(0.20)	
		Group 3	0.36(0.17)	0.44(0.26)	0.43(0.32)		0.40(0.26)	0.44(0.26)	
	Albumin	Total	4.65(0.23)	4.87(0.23)	4.86(0.28)	***	4.76(0.28)	4.87(0.23)	***
		Male	4.67(0.24)	4.91(0.23)	4.88(0.27)	***	4.78(0.28)	4.91(0.23)	***
		Female	4.63(0.22)	4.82(0.21)	4.84(0.29)	***	4.72(0.27)	4.82(0.21)	**
		Group 1	4.63(0.27)	4.79(0.22)	4.80(0.23)	***	4.71(0.27)	4.79(0.22)	**
		Group 2	4.59(0.19)	4.90(0.20)	4.75(0.24)	***	4.67(0.23)	4.90(0.20)	***
		Group 3	4.74(0.20)	4.92(0.24)	5.03(0.29)	***	4.89(0.29)	4.92(0.24)	
	SGOT (AST)	Total	15.72(3.57)	18.58(5.17)	17.59(5.11)	***	16.65(4.49)	18.58(5.17)	***
		Male	16.35(3.45)	19.51(5.25)	18.43(5.34)	***	17.62(4.85)	19.51(5.25)	***
		Female	14.92(3.59)	17.41(4.89)	16.54(4.65)	**	15.44(3.67)	17.41(4.89)	**
		Group 1	16.60(3.63)	19.53(6.28)	17.97(5.82)	**	17.28(4.86)	19.53(6.28)	***
		Group 2	15.38(3.20)	19.24(5.06)	17.10(4.47)	***	16.24(3.95)	19.24(5.06)	***
		Group 3	15.14(3.79)	16.93(3.51)	17.69(5.06)	**	16.41(4.61)	16.93(3.51)	
	SGPT (ALT)	Total	8.49(4.48)	11.00(7.86)	11.90(7.32)	***	10.19(6.29)	11.00(7.86)	
		Male	9.04(4.71)	12.02(8.12)	12.31(6.83)	***	11.16(6.94)	12.02(8.12)	*
		Female	7.79(4.14)	9.72(7.43)	11.38(7.96)	***	8.97(5.16)	9.72(7.43)	
		Group 1	8.90(4.57)	11.23(10.26)	10.30(6.93)		9.60(5.86)	11.23(10.26)	*
		Group 2	9.28(5.16)	12.45(7.92)	13.69(8.34)	**	11.48(7.22)	12.45(7.92)	
		Group 3	7.28(3.45)	9.31(3.87)	11.76(6.42)	***	9.52(5.59)	9.31(3.87)	
	Platelet	Total	272.75(67.65)	282.78(68.62)	288.07(65.45)	***	280.41(66.81)	282.78(68.62)	
		Male	262.27(61.68)	281.53(68.36)	284.35(60.26)	**	275.21(61.04)	281.53(68.36)	
		Female	279.64(74.74)	284.36(69.81)	292.74(71.97)		286.94(73.31)	284.36(69.81)	
		Group 1	287.10(73.99)	285.37(74.17)	282.43(73.81)		284.77(73.31)	285.37(74.17)	
		Group 2	245.38(53.63)	270.31(57.19)	279.66(58.92)	***	262.52(58.46)	270.31(57.19)	*
		Group 3	285.28(67.19)	292.59(73.44)	302.31(62.27)		293.79(64.78)	292.59(73.44)	
Mental Health	Resilience	Total	3.45(0.42)	3.72(0.55)	3.72(0.61)	***	3.59(0.54)	3.72(0.55)	***
		Male	3.51(0.41)	3.81(0.56)	3.72(0.58)	***	3.62(0.51)	3.81(0.56)	***
		Female	3.38(0.42)	3.62(0.53)	3.71(0.65)	***	3.55(0.57)	3.62(0.53)	
		Group 1	3.30(0.31)	3.26(0.36)	3.26(0.49)		3.56(0.55)	3.26(0.36)	
		Group 2	3.29(0.38)	3.44(0.39)	3.37(0.47)		3.54(0.52)	3.44(0.39)	***
		Group 3	3.27(0.37)	3.40(0.40)	3.52(0.46)	**	3.65(0.54)	3.40(0.40)	**
	Self-esteem	Total	2.64(0.33)	2.73(0.32)	2.74(0.34)	*	2.69(0.34)	2.73(0.32)	
		Male	2.71(0.33)	2.80(0.34)	2.77(0.36)		2.74(0.34)	2.80(0.34)	
		Female	2.55(0.32)	2.64(0.28)	2.69(0.32)	*	2.62(0.33)	2.64(0.28)	
		Group 1	2.83(0.47)	2.85(0.54)	2.86(0.54)		2.73(0.32)	2.85(0.54)	
		Group 2	3.01(0.45)	2.96(0.43)	2.99(0.46)		2.64(0.37)	2.96(0.43)	***
		Group 3	2.97(0.38)	2.98(0.34)	2.99(0.44)		2.70(0.34)	2.98(0.34)	
	Vigor	Total	3.05(1.28)	2.67(1.39)	2.63(1.47)	*	2.84(1.39)	2.67(1.39)	
		Male	3.02(1.30)	2.79(1.43)	2.75(1.51)		2.88(1.41)	2.79(1.43)	
		Female	3.09(1.26)	2.51(1.33)	2.47(1.42)	**	2.78(1.37)	2.51(1.33)	
		Group 1	2.87(1.32)	2.50(1.28)	2.65(1.37)		2.76(1.34)	2.50(1.28)	
		Group 2	3.06(1.21)	3.08(1.56)	2.66(1.59)		2.86(1.42)	3.08(1.56)	
		Group 3	3.22(1.31)	2.42(1.24)	2.57(1.49)		2.90(1.43)	2.42(1.24)	*
	Interpersonal Competency	Total	3.74(0.63)	3.79(0.70)	3.84(0.75)		3.79(0.69)	3.79(0.70)	
		Male	3.83(0.58)	3.86(0.68)	3.86(0.75)		3.84(0.67)	3.86(0.68)	
		Female	3.63(0.68)	3.70(0.73)	3.83(0.76)		3.73(0.73)	3.70(0.73)	
		Group 1	3.69(0.59)	3.69(0.82)	3.69(0.82)		3.68(0.71)	3.69(0.82)	
		Group 2	3.83(0.69)	3.80(0.67)	3.81(0.80)		3.81(0.74)	3.80(0.67)	
		Group 3	3.71(0.62)	3.90(0.68)	4.05(0.60)	**	3.88(0.63)	3.90(0.68)	
	Stress	Total	1.87(0.68)	1.82(0.70)	1.80(0.74)		1.84(0.71)	1.82(0.70)	
		Male	1.84(0.69)	1.84(0.68)	1.80(0.68)		1.82(0.68)	1.84(0.68)	
		Female	1.91(0.67)	1.80(0.73)	1.81(0.81)		1.86(0.74)	1.80(0.73)	
		Group 1	1.95(0.91)	2.01(0.90)	1.81(0.87)		1.98(0.75)	2.01(0.90)	
		Group 2	1.68(0.72)	1.48(0.71)	1.62(0.96)		1.77(0.74)	1.48(0.71)	
		Group 3	1.67(0.72)	1.59(0.69)	1.58(0.69)		1.76(0.61)	1.59(0.69)	

BP: blood pressure; RMSSD: root mean square of successive differences between normal heartbeats; Ln: natural logarithm (the spectral power data were log transformed); TP: total power; LF: power in the low frequency range; HF: power in the high frequency range; pNN50: percentage of successive RR intervals (interbeat intervals between all successive heartbeats) that differ by more than 50 ms; CD: cluster of differentiation molecule; IFN: interferon; TNF: tumor necrosis factor; IL: interleukin; IgA: immunoglobulin A; eNO: exhaled nitric oxide; 8-OHdG: 8-hydroxy-2-deoxyguanosine; d-ROMs: derivatives of reactive oxygen metabolites; BAP: biological antioxidant potential; BUN: blood urea nitrogen; SGOT: serum glutamic-oxaloacetic transaminase; AST: aspartate transaminase; SGPT: serum glutamic-pyruvic transaminase; ALT: alanine transaminase. * *p* < 0.05, ** *p* < 0.01, *** *p* < 0.001.

## Appendix B

**Table A2:** Result Table of Tukey’s Test for Post hoc Analysis.

Indicators		Group	Difference	Lower CI	Upper CI	*p*	
Physical Examination	Systolic BP	2–1	−5.6477	−10.5174	−0.7781	0.0183	*
		3–1	−5.0568	−9.9265	−0.1872	0.0398	*
		3–2	0.5909	−4.2788	5.4606	0.9559	
	Diastolic BP	2–1	−2.6023	−6.2004	0.9958	0.2053	
		3–1	−4.6705	−8.2686	−1.0723	0.0069	**
		3–2	−2.0682	−5.6663	1.5299	0.3663	
Heart Rate Variability	FATIGUE	2–1	0.1932	−0.2527	0.6390	0.5641	
		3–1	0.3864	−0.0595	0.8322	0.1042	
		3–2	0.1932	−0.2527	0.6390	0.5641	
	LnTP	2–1	−0.1298	−0.3071	0.0475	0.1977	
		3–1	−0.1560	−0.3333	0.0213	0.0972	
		3–2	−0.0263	−0.2036	0.1511	0.9351	
	LnHF	2–1	−0.4049	−0.7795	−0.0303	0.0306	*
		3–1	−0.3605	−0.7350	0.0141	0.0621	
		3–2	0.0444	−0.3301	0.4190	0.9578	
	Norm LF	2–1	6.8182	0.3605	13.2758	0.0357	*
		3–1	1.5909	−4.8667	8.0486	0.8306	
		3–2	−5.2273	−11.6849	1.2304	0.1384	
	Norm HF	2–1	−6.8182	−13.2758	−0.3605	0.0357	*
		3–1	−1.5909	−8.0486	4.8667	0.8306	
		3–2	5.2273	−1.2304	11.6849	0.1384	
	LnLF/HF	2–1	0.0711	0.0026	0.1397	0.0400	*
		3–1	0.0088	−0.0598	0.0773	0.9514	
		3–2	−0.0624	−0.1310	0.0062	0.0831	
	RMSSD	2–1	−14.4659	−24.9313	−4.0005	0.0036	**
		3–1	−14.7045	−25.1700	−4.2391	0.0030	**
		3–2	−0.2386	−10.7040	10.2268	0.9984	
Complete Blood Count	RBC	2–1	0.0095	−0.1582	0.1773	0.9901	
		3–1	−0.0797	−0.2474	0.0881	0.5030	
		3–2	−0.0892	−0.2570	0.0785	0.4229	
	Hemoglobin	2–1	−0.1341	−0.6838	0.4156	0.8336	
		3–1	−0.3205	−0.8702	0.2292	0.3560	
		3–2	−0.1864	−0.7361	0.3633	0.7039	
	Hematocrit	2–1	0.2500	−1.2020	1.7020	0.9133	
		3–1	−1.2080	−2.6600	0.2441	0.1241	
		3–2	−1.4580	−2.9100	−0.0059	0.0488	*
	Platelet	2–1	10.0341	−13.8645	33.9327	0.5840	
		3–1	15.3182	−8.5804	39.2168	0.2875	
		3–2	5.2841	−18.6145	29.1827	0.8611	
	WBC (Total)	2–1	−0.1445	−0.7572	0.4681	0.8434	
		3–1	0.8806	0.2679	1.4932	0.0023	**
		3–2	1.0251	0.4125	1.6377	0.0003	***
	Monocytes	2–1	−0.2341	−0.9002	0.4320	0.6857	
		3–1	−0.8864	−1.5525	−0.2203	0.0054	**
		3–2	−0.6523	−1.3184	0.0138	0.0564	
	Eosinophils	2–1	0.6852	−0.1179	1.4883	0.1115	
		3–1	0.1750	−0.6281	0.9781	0.8648	
		3–2	−0.5102	−1.3133	0.2929	0.2938	
	Basophils	2–1	−0.1966	−0.3168	−0.0764	0.0004	***
		3–1	−0.1443	−0.2645	−0.0241	0.0138	*
		3–2	0.0523	−0.0679	0.1725	0.5616	
Biochemical Examination	Serotonin	2–1	4.4818	−16.2303	25.1939	0.8665	
		3–1	22.9057	2.1936	43.6178	0.0261	*
		3–2	18.4239	−2.2882	39.1360	0.0925	
	25-(OH) Vitamin D	2–1	1.0739	−0.9514	3.0991	0.4250	
		3–1	1.6886	−0.3366	3.7139	0.1229	
		3–2	0.6148	−1.4105	2.6400	0.7545	
	1,25-(OH)3 Vitamin D	2–1	−13.7830	−19.8088	−7.7571	0.0000	***
		3–1	4.4682	−1.5577	10.4940	0.1895	
		3–2	18.2511	12.2253	24.2770	0.0000	***
	BAP	2–1	48.5568	−17.4669	114.5806	0.1947	
		3–1	−189.3068	−255.3306	−123.2831	0.0000	***
		3–2	−237.8636	−303.8874	−171.8399	0.0000	***
	Amylase	2–1	−0.8523	−9.7808	8.0763	0.9725	
		3–1	3.1136	−5.8149	12.0422	0.6897	
		3–2	3.9659	−4.9626	12.8945	0.5479	
	Protein	2–1	0.1443	0.0324	0.2562	0.0073	**
		3–1	0.1523	0.0404	0.2641	0.0043	**
		3–2	0.0080	−0.1039	0.1198	0.9846	
	Albumin	2–1	0.2205	0.1325	0.3084	0.0000	***
		3–1	0.2114	0.1235	0.2993	0.0000	***
		3–2	−0.0091	−0.0970	0.0788	0.9678	
	Creatinine	2–1	−0.0352	−0.0868	0.0163	0.2429	
		3–1	0.0045	−0.0470	0.0561	0.9765	
		3–2	0.0398	−0.0118	0.0913	0.1655	
	BUN	2–1	−1.1365	−2.1267	−0.1460	0.0199	*
		3–1	0.3636	−0.6267	1.3540	0.6625	
		3–2	1.5000	0.5096	2.4904	0.0012	**
	SGOT (AST)	2–1	2.8636	1.2026	4.5247	0.0002	***
		3–1	1.8750	0.2140	3.5360	0.0225	*
		3–2	−0.9886	−2.6497	0.6724	0.3408	
	SGPT (ALT)	2–1	2.5114	0.1228	4.8999	0.0367	*
		3–1	3.4091	1.0205	5.7977	0.0025	**
		3–2	0.8977	−1.4908	3.2863	0.6497	
	Uric Acid	2–1	−0.0511	−0.4302	0.3279	0.9458	
		3–1	0.2818	−0.0972	0.6609	0.1878	
		3–2	0.3330	−0.0461	0.7120	0.0980	
	Cholesterol (Total)	2–1	−4.1705	−12.4234	4.0825	0.4595	
		3–1	0.3750	−7.8780	8.6280	0.9937	
		3–2	4.5455	−3.7075	12.7984	0.3974	
	Glucose	2–1	−2.6250	−6.8645	1.6145	0.3122	
		3–1	10.1932	5.9537	14.4326	0.0000	***
		3–2	12.8182	8.5787	17.0576	0.0000	***
	Ca	2–1	0.2114	0.1073	0.3155	0.0000	***
		3–1	0.1125	0.0084	0.2166	0.0306	*
		3–2	−0.0989	−0.2030	0.0052	0.0667	
	Na	2–1	−1.3295	−1.9576	−0.7015	0.0000	***
		3–1	0.2273	−0.4008	0.8554	0.6704	
		3–2	1.5568	0.9287	2.1849	0.0000	***
	Cl	2–1	0.0455	−0.6537	0.7446	0.9871	
		3–1	0.5455	−0.1537	1.2446	0.1590	
		3–2	0.5000	−0.1992	1.1992	0.2126	
Immuno- serological Examination	CD16+CD56 (count)	2–1	−38.0581	−94.7144	18.5982	0.2546	
		3–1	3.0067	−53.6497	59.6630	0.9914	
		3–2	41.0648	−15.5916	97.7211	0.2039	
	CD16+CD56 (WB)	2–1	−1.6455	−3.6798	0.3889	0.1388	
		3–1	−1.6432	−3.6775	0.3911	0.1396	
		3–2	0.0023	−2.0320	2.0366	1.0000	
	IL-8 M_Multiplex	2–1	53.1813	34.8497	71.5129	0.0000	***
		3–1	0.4193	−17.9123	18.7509	0.9984	
		3–2	−52.7620	−71.0937	−34.4304	0.0000	***
	TNF-α HS M_Multiplex	2–1	−2.0337	−3.3696	−0.6977	0.0012	**
		3–1	−2.3386	−3.6745	−1.0026	0.0001	***
		3–2	−0.3049	−1.6408	1.0311	0.8527	
	IFN-γ HS M_Multiplex	2–1	0.7074	−5.9449	7.3597	0.9660	
		3–1	3.0468	−3.6054	9.6991	0.5276	
		3–2	2.3394	−4.3129	8.9917	0.6854	
	CRP	2–1	0.0730	−0.0068	0.1527	0.0806	
		3–1	0.0223	−0.0574	0.1020	0.7877	
		3–2	−0.0507	−0.1304	0.0290	0.2933	
Urine	pH (RU)	2–1	0.0682	−0.2054	0.3418	0.8270	
		3–1	0.3409	0.0673	0.6145	0.0101	*
		3–2	0.2727	−0.0009	0.5463	0.0510	
	Flow cytometry	2–1	0.0114	−0.0834	0.1061	0.9569	
	(E.P. CELL)	3–1	0.1136	0.0189	0.2084	0.0140	*
		3–2	0.1023	0.0075	0.1971	0.0309	*
	Flow cytometry	2–1	0.1477	0.0475	0.2480	0.0017	**
	(OTHER)	3–1	−0.0114	−0.1116	0.0889	0.9614	
		3–2	−0.1591	−0.2593	−0.0589	0.0007	***
Psychological Examination	Resilience	2–1	0.2710	0.0822	0.4598	0.0024	**
		3–1	0.2660	0.0772	0.4548	0.0029	**
		3–2	−0.0051	−0.1939	0.1838	0.9978	
	Self-esteem	2–1	0.0920	−0.0261	0.2102	0.1597	
		3–1	0.0955	−0.0227	0.2136	0.1394	
		3–2	0.0034	−0.1147	0.1215	0.9975	
	Vigor	2–1	−0.3818	−0.8718	0.1082	0.1597	
		3–1	−0.4205	−0.9105	0.0696	0.1088	
		3–2	−0.0386	−0.5287	0.4514	0.9811	

1: Pre-treatment assessment, 2: Post-treatment assessment, 3: Follow up assessment. BP: blood pressure; Ln: natural logarithm (the spectral power data were log transformed); TP: total power; HF: power in the high frequency range; LF: power in the low frequency range; RMSSD: root mean square of successive differences between normal heartbeats; RBC: red blood cells; WBC: white blood cells; BAP: biological antioxidant potential; BUN: blood urea nitrogen; SGOT: serum glutamic-oxaloacetic transaminase; AST: aspartate transaminase; SGPT: serum glutamic-pyruvic transaminase; ALT: alanine transaminase; Ca: calcium; Na: sodium; Cl: chlorine; CD: cluster of differentiation molecule; IL: interleukin; TNF: tumor necrosis factor; IFN: interferon; CRP: c-reactive protein; pH: potential hydrogen; RU: ratio urine; E.P. Cell: epithelial cell. * *p* < 0.05, ** *p* < 0.01, *** *p* < 0.001.

## Appendix C

**Table A3:** Result Table of RM ANOVA by Groups.

Indices		Group	N	Pre-Treatment Assessment	Post- Treatment Assessment	Follow-Up Assessment	F	*p*	
				M(SD)	M(SD)	M(SD)			
Physiological	Systolic BP	1	30	127.97(15.27)	119.87(12.68)	116.16(11.09)	14.619	0.000	***
Indices		2	29	129.00(15.26)	116.45(14.30)	125.21(12.93)	100.612	0.000	***
		3	29	113.62(10.41)	117.48(11.14)	114.59(11.52)	1.857	0.166	
	Diastolic BP	1	30	74.84(13.72)	72.87(7.64)	70.06(11.61)	2.209	0.119	
		2	29	79.86(10.12)	72.45(11.28)	74.10(8.99)	9.897	0.000	***
		3	29	74.10(9.45)	75.55(7.81)	70.41(7.95)	6.362	0.003	**
	Pulse	1	30	74.94(12.46)	78.84(10.65)	80.03(9.53)	3.118	0.051	
		2	29	74.17(9.24)	72.24(11.39)	75.83(10.59)	2.153	0.126	
		3	29	81.86(11.03)	75.76(9.09)	78.33(12.26)	3.563	0.035	*
	Body	1	30	36.69(0.30)	36.61(0.35)	36.70(0.31)	1.224	0.301	
	Temperature	2	29	36.67(0.28)	36.66(0.26)	36.64(0.36)	0.104	0.902	
		3	29	36.83(0.37)	36.73(0.28)	36.72(0.45)	1.443	0.245	
	eNO	1	30	13.42(8.66)	16.23(12.12)	13.52(8.23)	3.245	0.046	*
		2	29	22.21(29.68)	22.43(29.44)	18.68(22.15)	3.244	0.047	*
		3	29	13.17(9.48)	15.14(14.92)	16.59(15.77)	2.064	0.136	
	HR	1	30	75.77(12.89)	83.33(10.74)	83.73(10.85)	9.542	0.000	***
		2	29	77.72(10.65)	78.52(10.86)	79.03(10.68)	0.276	0.760	
		3	29	84.00(8.90)	79.97(7.79)	79.55(7.77)	3.851	0.027	*
	SDNN	1	30	61.90(29.48)	49.70(26.06)	53.13(24.22)	3.357	0.042	*
		2	29	71.14(34.35)	68.45(45.17)	59.45(29.91)	1.139	0.327	
		3	29	55.10(27.26)	58.83(27.98)	49.14(17.31)	1.442	0.245	
	RMSSD	1	30	60.10(31.50)	34.93(20.42)	39.83(20.70)	14.921	0.000	***
		2	29	65.69(45.66)	51.90(33.04)	49.03(30.53)	3.113	0.052	
		3	29	47.45(28.53)	43.38(24.85)	40.45(15.91)	0.694	0.504	
	HRV Index	1	30	11.81(3.16)	10.58(2.83)	10.31(3.23)	4.989	0.010	*
		2	29	12.02(3.72)	12.23(3.73)	11.62(4.03)	0.355	0.703	
		3	29	11.01(3.74)	11.60(3.22)	11.24(3.35)	0.347	0.708	
	pNN50	1	30	71.43(18.08)	59.33(17.46)	59.00(20.49)	8.216	0.001	**
		2	29	65.55(16.32)	67.03(16.92)	64.24(16.36)	0.649	0.527	
		3	29	64.17(12.10)	62.28(15.71)	69.76(11.95)	3.338	0.043	*
	LnTP	1	30	7.23(0.57)	6.81(0.37)	6.89(0.47)	11.107	0.000	***
		2	29	7.20(0.56)	7.18(0.67)	7.05(0.44)	1.203	0.308	
		3	29	6.93(0.45)	7.01(0.39)	6.98(0.39)	0.405	0.669	
	LnVLF	1	30	6.70(0.39)	6.47(0.33)	6.49(0.30)	7.181	0.002	**
		2	29	6.63(0.37)	6.33(0.31)	6.59(0.28)	0.226	0.799	
		3	29	6.48(0.24)	6.57(0.27)	6.57(0.22)	1.704	0.191	
	LnLF	1	30	5.35(1.09)	4.78(0.82)	4.92(1.15)	3.624	0.033	*
		2	29	5.52(1.19)	5.53(1.33)	5.11(1.18)	1.571	0.217	
		3	29	5.04(1.10)	5.38(0.77)	4.99(1.09)	2.486	0.092	
	LnHF	1	30	5.41(1.04)	4.47(0.81)	4.60(1.04)	14.700	0.000	***
		2	29	5.14(1.23)	4.96(1.18)	4.86(1.16)	0.988	0.379	
		3	29	4.80(1.15)	4.73(0.89)	4.82(0.86)	0.138	0.871	
	Norm LF	1	30	48.57(18.60)	56.07(18.96)	57.43(14.11)	3.226	0.047	*
		2	29	58.21(19.97)	62.31(17.56)	55.38(21.00)	1.496	0.233	
		3	29	55.28(16.24)	64.10(14.40)	53.76(20.65)	5.262	0.008	**
	Norm HF	1	30	51.43(18.60)	43.93(18.96)	42.57(14.11)	3.226	0.047	*
		2	29	41.79(19.97)	37.69(17.56)	44.62(21.00)	1.496	0.233	
		3	29	44.72(16.24)	35.90(14.40)	46.24(20.65)	5.262	0.008	**
	LnLF/HF	1	30	1.00(0.17)	1.09(0.22)	1.08(0.14)	2.978	0.059	
		2	29	1.09(0.20)	1.13(0.18)	1.07(0.23)	1.153	0.323	
		3	29	1.07(0.19)	1.16(0.18)	1.05(0.21)	4.302	0.018	*
	PSI	1	30	4.83(0.63)	5.03(0.62)	5.00(0.67)	2.090	0.133	
		2	29	4.67(0.80)	4.72(0.70)	4.85(0.80)	0.674	0.514	
		3	29	4.92(0.72)	4.82(0.62)	4.98(0.52)	0.749	0.478	
	STRESS	1	30	4.43(2.30)	5.70(1.74)	5.60(2.42)	4.667	0.013	*
		2	29	4.10(2.54)	4.17(2.42)	4.72(2.39)	0.946	0.394	
		3	29	5.24(2.43)	4.52(1.99)	5.24(2.36)	1.886	0.161	
	HEALTH	1	30	78.87(6.49)	76.00(6.92)	75.13(9.24)	2.573	0.085	
		2	29	80.03(8.49)	79.14(7.93)	77.5(8.73)	1.501	0.230	
		3	29	77.59(9.23)	80.34(6.56)	77.55(7.59)	2.145	0.127	
	FATIGUE	1	30	2.30(1.02)	2.97(1.03)	2.97(1.40)	4.411	0.016	*
		2	29	2.10(1.37)	2.34(1.20)	2.52(1.45)	1.295	0.282	
		3	29	2.66(1.34)	2.31(1.04)	2.72(1.25)	2.188	0.122	
	RBC	1	30	4.80(0.41)	4.88(0.43)	4.76(0.40)	9.513	0.000	***
		2	29	4.95(0.55)	4.91(0.50)	4.84(0.50)	4.633	0.014	*
		3	29	4.78(0.49)	4.78(0.48)	4.69(0.47)	3.719	0.030	*
	Hb	1	30	13.40(1.55)	13.46(1.54)	13.17(1.54)	8.489	0.001	**
		2	29	14.32(1.59)	13.99(1.44)	13.86(1.54)	9.834	0.000	***
		3	29	13.80(1.57)	13.66(1.57)	13.52(1.47)	4.202	0.020	*
	Hct	1	30	42.31(3.97)	43.09(4.06)	40.89(3.91)	36.392	0.000	***
		2	29	44.47(4.24)	44.21(4.00)	41.85(3.92)	33.646	0.000	***
		3	29	41.44(4.08)	41.65(3.84)	41.86(4.07)	1.135	0.329	
	Platelet	1	30	287.10(73.99)	285.37(74.17)	282.43(73.81)	0.308	0.736	
		2	29	245.38(53.63)	270.31(57.19)	279.66(58.92)	38.796	0.000	***
		3	29	285.28(67.19)	292.59(73.44)	302.31(62.27)	2.886	0.064	
	WBC	1	30	7.08(1.39)	6.00(1.59)	6.89(1.73)	8.786	0.000	***
		2	29	5.00(1.40)	6.10(1.34)	7.69(1.78)	56.835	0.000	***
		3	29	7.36(1.48)	6.94(1.86)	7.54(1.73)	3.472	0.038	*
	Neutrophil	1	30	56.92(9.55)	50.24(9.54)	53.68(8.69)	10.437	0.000	***
	Seg.	2	29	46.86(12.17)	51.46(9.88)	53.25(9.87)	7.835	0.001	**
		3	29	52.53(6.88)	54.09(9.12)	53.28(8.36)	0.436	0.649	
	Lymphocyte	1	30	32.66(8.34)	37.59(8.77)	35.82(7.12)	6.755	0.002	**
		2	29	39.41(10.74)	36.59(9.01)	35.98(9.58)	3.283	0.045	*
		3	29	36.48(6.68)	34.19(8.58)	35.44(7.81)	1.181	0.315	
	Monocyte	1	30	7.81(1.82)	8.32(1.88)	7.26(1.43)	4.921	0.011	*
		2	29	9.61(2.28)	8.12(1.82)	7.64(1.36)	14.476	0.000	***
		3	29	7.96(1.86)	8.22(1.82)	7.81(2.03)	1.070	0.350	
	Eosinophil	1	30	1.88(1.74)	3.11(2.52)	2.48(1.89)	10.419	0.000	***
		2	29	2.90(2.17)	3.14(2.13)	2.30(1.55)	6.426	0.003	**
		3	29	2.27(1.46)	2.82(2.19)	2.77(3.86)	0.823	0.444	
	Basophil	1	30	0.74(0.33)	0.74(0.33)	0.75(0.35)	0.036	0.965	
		2	29	1.22(0.37)	0.69(0.25)	0.83(0.32)	26.099	0.000	***
		3	29	0.76(0.27)	0.69(0.29)	0.70(0.30)	1.817	0.172	
	IL-4 HS	1	30	69.28(40.10)	70.40(48.18)	83.04(44.94)	6.215	0.004	**
	M_Multiplex	2	29	100.70(67.04)	77.72(48.14)	88.74(60.56)	10.753	0.000	***
		3	29	84.48(62.96)	97.70(77.87)	64.24(67.84)	32.681	0.000	***
	IL-8	1	30	12.12(9.71)	118.79(107.18)	17.33(11.13)	31.546	0.000	***
	M Multiplex	2	29	16.81(11.16)	78.00(56.71)	13.40(12.56)	39.377	0.000	***
		3	29	32.91(36.96)	22.76(24.52)	32.21(43.37)	3.194	0.049	*
	TNF-α HS	1	30	16.11(2.99)	14.66(3.52)	15.50(3.56)	3.988	0.024	*
	M Multiplex	2	29	17.66(3.81)	12.84(3.36)	13.22(2.57)	66.652	0.000	***
		3	29	15.74(4.34)	15.88(3.83)	13.71(4.52)	6.699	0.002	**
	IFN-γ HS	1	30	28.44(11.72)	29.47(13.63)	36.11(15.70)	19.251	0.000	***
	M Multiplex	2	29	32.47(12.38)	26.74(9.21)	33.75(16.38)	8.188	0.001	**
		3	29	34.64(21.67)	41.45(31.81)	34.67(23.20)	4.495	0.015	*
	CD16+	1	30	301.49(152.92)	318.63(159.84)	364.00(172.09)	5.228	0.008	**
	CD56 (count)	2	29	284.50(155.22)	250.83(108.97)	268.57(204.46)	0.441	0.645	
		3	29	365.90(151.71)	266.35(140.01)	326.28(154.44)	6.856	0.002	**
	CD16+	1	30	13.53(6.24)	14.66(6.35)	15.01(5.97)	2.034	0.140	
	CD56 (WB)	2	29	15.27(7.36)	11.48(4.26)	9.96(5.84)	12.399	0.000	***
		3	29	13.62(4.18)	11.25(4.12)	12.42(4.73)	4.265	0.019	*
	CRP	1	30	0.08(0.18)	0.16(0.35)	0.14(0.36)	0.996	0.375	
		2	29	0.04(0.07)	0.09(0.22)	0.04(0.09)	1.984	0.147	
		3	29	0.06(0.11)	0.14(0.29)	0.06(0.10)	2.803	0.069	
	IgA (S)	1	30	178.43(54.04)	180.78(52.18)	178.06(52.73)	1.366	0.263	
		2	29	182.48(57.38)	181.32(54.42)	175.86(53.26)	5.108	0.009	**
		3	29	190.25(82.18)	187.31(78.61)	183.42(73.72)	0.120	0.887	
	Protein,	1	30	7.21(0.29)	7.29(0.28)	7.25(0.26)	1.767	0.180	
	total	2	29	7.08(0.29)	7.31(0.34)	7.27(0.34)	11.232	0.000	***
		3	29	7.32(0.25)	7.44(0.29)	7.54(0.35)	6.811	0.002	**
	Albumin	1	30	4.63(0.27)	4.79(0.22)	4.80(0.23)	14.911	0.000	***
		2	29	4.59(0.19)	4.90(0.20)	4.75(0.24)	33.338	0.000	***
		3	29	4.74(0.20)	4.92(0.24)	5.03(0.29)	20.248	0.000	***
	Creatinine	1	30	0.73(0.16)	0.73(0.15)	0.76(0.16)	2.027	0.141	
		2	29	0.78(0.15)	0.75(0.12)	0.79(0.13)	4.039	0.023	*
		3	29	0.78(0.15)	0.70(0.13)	0.76(0.14)	8.790	0.000	***
	BUN	1	30	11.23(2.86)	9.97(2.85)	13.27(3.05)	27.655	0.000	***
		2	29	11.90(3.02)	10.93(2.51)	11.62(2.51)	1.985	0.147	
		3	29	13.41(2.98)	12.24(2.20)	12.69(1.91)	2.736	0.073	
	Bilirubin,	1	30	0.47(0.26)	0.51(0.25)	0.52(0.28)	0.546	0.582	
	total	2	29	0.54(0.24)	0.48(0.20)	0.51(0.28)	1.304	0.280	
		3	29	0.36(0.17)	0.44(0.26)	0.43(0.32)	3.031	0.056	
	AST (SGOT)	1	30	16.60(3.63)	19.53(6.28)	17.97(5.82)	5.443	0.007	**
		2	29	15.38(3.20)	19.24(5.06)	17.10(4.47)	11.640	0.000	***
		3	29	15.14(3.79)	16.93(3.51)	17.69(5.06)	5.602	0.006	**
	ALT (SGPT)	1	30	8.90(4.57)	11.23(10.26)	10.30(6.93)	2.808	0.069	
		2	29	9.28(5.16)	12.45(7.92)	13.69(8.34)	6.618	0.003	**
		3	29	7.28(3.45)	9.31(3.87)	11.76(6.42)	17.924	0.000	***
	ALP	1	30	154.87(113.04)	156.83(117.04)	152.83(112.45)	1.148	0.324	
		2	29	133.55(92.53)	136.41(94.25)	128.17(86.82)	7.051	0.002	**
		3	29	118.76(67.82)	116.83(64.60)	115.41(60.99)	0.229	0.796	
	Uric Acid	1	30	5.20(1.09)	5.34(1.11)	5.92(1.08)	19.052	0.000	***
		2	29	5.60(1.10)	5.45(1.14)	5.83(1.02)	5.567	0.006	**
		3	29	5.51(0.91)	5.36(1.04)	5.39(1.08)	0.893	0.415	
	Cholesterol,	1	30	144.20(20.05)	137.60(20.94)	141.27(20.82)	5.042	0.010	*
	total	2	29	148.10(26.69)	148.90(28.00)	153.21(26.17)	2.237	0.116	
		3	29	151.03(18.96)	144.41(20.26)	150.10(24.15)	4.130	0.021	*
	Glucose (S)	1	30	83.77(12.40)	80.80(9.48)	97.63(13.27)	25.270	0.000	***
		2	29	88.62(9.33)	87.76(8.96)	98.93(10.60)	12.857	0.000	***
		3	29	86.90(13.63)	82.86(9.76)	93.17(16.51)	4.105	0.022	*
	Inorganic-	1	30	4.13(0.84)	3.99(0.69)	4.35(0.62)	8.244	0.001	**
	phosphorus	2	29	3.81(0.42)	4.16(0.59)	4.00(0.51)	8.020	0.001	**
		3	29	4.38(0.58)	4.30(0.46)	4.28(0.48)	0.499	0.610	
	Calcium	1	30	9.54(0.28)	9.79(0.24)	9.54(0.20)	19.572	0.000	***
		2	29	9.76(0.27)	9.88(0.37)	9.87(0.24)	2.626	0.081	
		3	29	9.56(0.25)	9.83(0.31)	9.79(0.30)	14.308	0.000	***
	Na (Sodium)	1	30	141.33(1.99)	140.60(1.81)	141.53(1.55)	4.592	0.014	*
		2	29	141.76(1.41)	139.48(1.12)	140.45(1.53)	30.133	0.000	***
		3	29	141.34(1.70)	140.34(1.90)	143.14(1.57)	26.990	0.000	***
	K	1	30	4.22(0.36)	4.04(0.37)	4.12(0.35)	4.374	0.017	*
	(Potassium)	2	29	4.34(0.25)	4.27(0.28)	4.29(0.26)	1.183	0.314	
		3	29	4.07(0.31)	4.16(0.30)	4.28(0.24)	7.040	0.002	**
	Cl (Chlorine)	1	30	100.97(2.51)	101.17(2.29)	101.60(2.44)	1.576	0.215	
		2	29	101.52(1.64)	100.93(1.41)	100.31(1.28)	9.509	0.000	***
		3	29	100.66(1.49)	101.17(1.69)	102.86(1.64)	23.843	0.000	***
	Cortisol	1	30	5.81(2.78)	6.23(2.54)	5.55(1.96)	0.730	0.486	
	(CIA)	2	29	6.74(2.26)	6.34(2.78)	7.32(3.20)	1.412	0.252	
		3	29	6.55(2.61)	7.30(2.55)	6.94(3.19)	0.667	0.517	
	Serotonin	1	30	152.26(44.52)	138.55(41.34)	177.58(50.76)	29.350	0.000	***
	(HPLC)	2	29	155.76(52.38)	186.54(79.57)	192.77(74.88)	9.905	0.000	***
		3	29	129.68(45.01)	126.67(44.53)	135.98(46.78)	0.737	0.483	*
	1,25-(OH)2	1	30	29.18(16.63)	8.53(7.23)	23.37(16.07)	31.386	0.000	***
	vitamin D	2	29	27.86(14.34)	23.16(22.31)	22.97(12.12)	1.314	0.277	
		3	29	25.69(16.41)	9.93(8.49)	50.16(15.25)	79.349	0.000	***
	25-(OH)	1	30	21.89(7.08)	21.31(6.59)	21.72(6.53)	1.931	0.154	
	vitamin D	2	29	19.62(4.47)	21.15(5.02)	21.63(4.59)	16.818	0.000	***
		3	29	18.52(6.11)	20.86(5.21)	21.81(4.97)	15.277	0.000	***
	d-ROMs	1	30	314.60(62.27)	320.30(66.39)	319.83(65.82)	0.640	0.531	
		2	29	314.24(72.53)	314.90(68.87)	307.45(73.36)	1.054	0.355	
		3	29	304.07(60.55)	303.00(59.59)	317.17(56.02)	3.484	0.037	*
	BAP	1	30	2138.30(140.26)	2171.33(166.45)	1840.37(131.38)	139.420	0.000	***
		2	29	2130.21(173.30)	2176.17(148.53)	1850.76(204.81)	85.761	0.000	***
		3	29	2132.97(161.29)	2200.17(174.47)	2146.17(202.37)	2.739	0.073	
	Amylase (S)	1	30	74.23(31.09)	72.50(26.43)	75.70(31.16)	0.624	0.539	
		2	29	62.00(20.77)	62.14(15.72)	66.28(27.20)	2.009	0.144	
		3	29	64.93(20.30)	64.00(20.80)	68.59(26.25)	3.143	0.051	
	α-Amylase	1	30	16.87(13.41)	12.34(10.65)	24.57(15.83)	3.261	0.056	
	(Saliva)	2	29	8.90(9.64)	8.85(7.69)	7.56(7.08)	0.121	0.887	
		3	29	18.44(13.25)	8.55(5.71)	7.51(7.47)	10.077	0.000	***
	Secretory	1	30	19.75(16.11)	117.79(102.80)	70.54(55.36)	7.401	0.004	**
	IgA (Saliva)	2	29	34.25(30.46)	65.83(51.01)	91.33(64.76)	12.037	0.000	***
		3	29	78.76(66.31)	87.63(84.92)	115.48(57.67)	3.110	0.054	
	Cortisol	1	30	0.09(0.09)	0.10(0.03)	0.10(0.05)	0.086	0.918	
	(Saliva)	2	29	0.09(0.05)	0.07(0.04)	0.15(0.09)	8.906	0.001	**
		3	29	0.10(0.09)	0.11(0.07)	0.10(0.05)	0.071	0.931	
	Creatinine	1	30	145.86(70.55)	166.46(64.90)	174.75(53.43)	1.891	0.160	
	(RU)	2	29	152.81(91.06)	177.76(89.02)	139.09(70.60)	2.869	0.065	
		3	29	178.62(85.26)	181.72(60.52)	181.18(86.84)	0.018	0.982	
	pH	1	30	6.15(0.87)	6.15(0.72)	6.63(0.63)	4.517	0.015	*
	(RU)	2	29	5.88(0.88)	6.12(0.66)	6.16(0.90)	1.358	0.266	
		3	29	6.12(0.82)	6.09(0.58)	6.38(0.76)	1.968	0.149	
	Specific	1	30	1.02(0.01)	1.02(0.01)	1.03(0.00)	5.286	0.008	**
	Gravity (RU)	2	29	1.02(0.01)	1.02(0.01)	1.02(0.01)	1.638	0.203	
		3	29	1.02(0.01)	1.03(0.01)	1.02(0.01)	1.933	0.154	
	8-OHdG (U)	1	30	11.73(5.95)	14.69(6.59)	12.47(6.52)	2.467	0.094	
		2	29	11.60(6.78)	12.95(6.56)	11.43(5.87)	0.794	0.457	
		3	29	13.61(8.08)	11.73(3.91)	11.42(5.91)	1.336	0.271	
Psychological	Resilience	1	30	3.30(0.31)	3.26(0.36)	3.26(0.49)	0.244	0.784	
Indices		2	29	3.29(0.38)	3.44(0.39)	3.37(0.47)	2.212	0.119	
		3	29	3.27(0.37)	3.40(0.40)	3.52(0.46)	6.630	0.003	**
	Interpersonal	1	30	3.69(0.59)	3.69(0.82)	3.69(0.82)	0.000	1.000	
	Competency	2	29	3.83(0.69)	3.80(0.67)	3.81(0.80)	0.046	0.956	
		3	29	3.71(0.62)	3.90(0.68)	4.05(0.60)	5.216	0.008	**
	Self-esteem	1	30	2.83(0.47)	2.85(0.54)	2.86(0.54)	0.121	0.886	
		2	29	3.01(0.45)	2.96(0.43)	2.99(0.46)	0.240	0.787	
		3	29	2.97(0.38)	2.98(0.34)	2.99(0.44)	0.044	0.957	
	Stress	1	30	1.95(0.91)	2.01(0.90)	1.81(0.87)	2.015	0.143	
		2	29	1.68(0.72)	1.48(0.71)	1.62(0.96)	1.067	0.351	
		3	29	1.67(0.72)	1.59(0.69)	1.58(0.69)	0.314	0.732	
	Vigor	1	30	2.87(1.32)	2.50(1.28)	2.65(1.37)	0.953	0.392	
		2	29	3.06(1.21)	3.08(1.56)	2.66(1.59)	1.008	0.371	
		3	29	3.22(1.31)	2.42(1.24)	2.57(1.49)	5.930	0.005	

Group 1: 1-week-after group; Group 2: 2-weeks-after group; Group 3: 4-weeks-after group; BP: blood pressure; eNO: exhaled nitric oxide; HR: heart rate; SDNN: standard deviation of normal-to-normal intervals; RMSSD: root mean square of successive differences between normal heartbeats; HRV: heart rate variability; pNN50: percentage of successive RR intervals (interbeat intervals between all successive heartbeats) that differ by more than 50 ms; Ln: natural logarithm (the spectral power data were log transformed); TP: total power; VLF: power in the very low frequency range; LF: power in the low frequency range; HF: power in the high frequency range; PSI: physiological strain index; RBC: red blood cells; Hb: hemoglobin; Hct: hematocrit; WBC: white blood cells; Seg.: segmented; IL: interleukin; TNF: tumor necrosis factor; IFN: interferon; CD: cluster of differentiation molecule; CRP: c-reactive protein; IgA: immunoglobulin A; BUN: blood urea nitrogen; AST: aspartate transaminase; SGOT: serum glutamic-oxaloacetic transaminase; ALT: alanine transaminase; SGPT: serum glutamic-pyruvic transaminase; ALP: alkaline phosphatase; d-ROMs: derivatives of reactive oxygen metabolites; BAP: biological antioxidant potential; pH: potential hydrogen; 8-OHdG: 8-hydroxy-2-deoxyguanosine. * *p* < 0.05, ** *p* < 0.01, *** *p* < 0.001.

## Appendix D

**Table A4:** Result Table of Participant’s Allergy Status.

Classification	Allergens	Results	Groups			Total
			Group 1	Group 2	Group 3	
Plants	Grass	Negative	31	26	28	85
		Positive	2	3	1	6
	Tree 1 (bloom in early spring)	Negative	28	28	28	84
		Positive	5	1	0	6
	Tree 2 (bloom in late spring)	Negative	28	27	28	83
		Positive	5	2	0	7
	Mugwort	Negative	31	29	29	89
		Positive	2	0	0	2
	Birch	Negative	28	27	28	83
		Positive	4	2	0	6
	Nettle	Negative	31	27	28	86
		Positive	2	2	0	4
	Oak	Negative	28	27	28	83
		Positive	5	1	0	6
	Alder	Negative	29	28	28	85
		Positive	4	1	0	5
	Humulus Japonicus	Negative	28	28	28	84
		Positive	5	1	0	6
	Bermuda grass	Negative	30	28	29	87
		Positive	3	1	0	4
	Orchard grass	Negative	30	27	29	86
		Positive	3	2	0	5
	Poplar	Negative	30	29	29	88
		Positive	3	0	0	3
	Ragweed	Negative	31	29	29	89
		Positive	2	0	0	2
	Engl. Plantain	Negative	28	28	29	85
		Positive	5	1	0	6
	Timothy grass	Negative	32	26	29	87
		Positive	1	3	0	4
	Meadow Fescue	Negative	31	27	29	87
		Positive	2	2	0	4
	Total Allergy Reaction Counts		53	22	1	76
Molds	Mold 1	Negative	33	28	29	90
		Positive	0	1	0	1
	Mold 2	Negative	32	27	29	88
		Positive	1	2	0	3
	Alternaria	Negative	31	26	25	82
		Positive	2	3	3	8
	Aspergillus	Negative	31	28	29	88
		Positive	2	1	0	3
	Candida	Negative	33	29	29	91
		Positive	0	0	0	0
	Cladosporium	Negative	32	27	29	88
		Positive	1	2	0	3
	Penicillium	Negative	32	27	29	88
		Positive	1	2	0	3
	Total Allergy Reaction Counts		7	11	3	21
Animals, Pests, and Dust Mites	Dog	Negative	31	29	29	89
		Positive	2	0	0	2
	Cat	Negative	28	28	27	83
		Positive	5	1	2	8
	D. Farinae	Negative	24	19	26	69
		Positive	9	10	3	22
	D. pteronys	Negative	21	19	24	64
		Positive	12	10	5	27
	Tyrophagus	Negative	28	24	29	81
		Positive	5	5	0	10
	Cockroach	Negative	31	24	28	83
		Positive	2	4	1	7
	Total Allergy Reaction Counts		35	30	11	76

Group 1: 1-week-after group; Group 2: 2-weeks-after group; Group 3: 4-weeks-after group.
